# Protein Contribution to Plant Salinity Response and Tolerance Acquisition

**DOI:** 10.3390/ijms14046757

**Published:** 2013-03-26

**Authors:** Klára Kosová, Ilja T. Prášil, Pavel Vítámvás

**Affiliations:** Crop Research Institute, Department of Genetics and Plant Breeding, Drnovská 507/73, 161 06 Prague 6, Czech Republic; E-Mails: prasil@vurv.cz (I.T.P.); vitamvas@vurv.cz (P.V.)

**Keywords:** salinity response, proteome, glycophyte, halophyte, comparative proteomics

## Abstract

The review is focused on plant proteome response to salinity with respect to physiological aspects of plant salt stress response. The attention is paid to both osmotic and ionic effects of salinity stress on plants with respect to several protein functional groups. Therefore, the role of individual proteins involved in signalling, changes in gene expression, protein biosynthesis and degradation and the resulting changes in protein relative abundance in proteins involved in energy metabolism, redox metabolism, stressand defence-related proteins, osmolyte metabolism, phytohormone, lipid and secondary metabolism, mechanical stress-related proteins as well as protein posttranslational modifications are discussed. Differences between salt-sensitive (glycophytes) and salt-tolerant (halophytes) plants are analysed with respect to differential salinity tolerance. In conclusion, contribution of proteomic studies to understanding plant salinity tolerance is summarised and discussed.

## 1. Introduction

Soil salinity is caused by an increased activity of soluble salts. As saline soils, soils with electrical conductivity higher than 4 dS m^−1^ are generally considered [1]. In most proteomic papers as well as in nature, NaCl represents the major soluble salt causing soil salinity (sodicity). Salinity represents a strong limitation of agricultural production worldwide, especially in arid and semi-arid regions where salts from basal rocks come into the upper layer of soil due to a prevalence of water evaporation over precipitation (low annual rainfall). It is estimated that about 7% of world agricultural land is affected by salinity and that this number could increase up to 20% in the future due to land salinization as a consequence of an artificial irrigation and an unsuitable land management. Regarding irrigated soils which contribute to roughly one third of the global food production, it is estimated that nearly one half of the total area of irrigated land could be adversely affected by salinization [2,3].

Salinity reveals adverse effects on plants. Elevated levels of salt ions in soil solution surrounding plant roots induce an imbalance in water potential between plant root cells and ambient soil solution and result in cellular dehydration. In addition, elevated salts lead to a passive salt ion penetration via plasma membrane and to an accumulation of salt ions in cell cytoplasm which can lead to inhibition of intracellular enzyme activity [3]. The decrease in osmotic potential, *i.e.*, an osmotic effect, is common to all abiotic stresses associated with dehydration, *i.e.*, salinity, drought, enhanced evaporation, low temperature stress, mechanical wounding. The increase in salt ion concentrations is specific to salinity stress. Plants cope with osmotic effect by the mechanisms of osmotic adjustment, *i.e.*, decreasing cellular osmotic potential. In addition, plants cope with increased ion concentrations either via salt ion exclusion from the cells or salt ion compartmentation in the intracellular compartments (accumulation in vacuoles). Moreover, plants also have to cope with oxidative stress. These adverse stress factors result in an enhanced stomata closure, a decreased photosynthesis rate and a reduced plant growth. The plant ability to cope with stress underlies plant salinity tolerance [4].

Plant salinity tolerance can be assessed either as a reduction in relative plant growth rate after a prolonged exposure to a given concentration of salt or as a plant survival rate after a treatment with a defined concentration of salt [2].

Plants differ in their ability to cope with these adverse factors. According to tolerance to salinity, plants can be basically divided into two groups: glycophytes and halophytes. Halophytes can generally tolerate high salt concentrations and they can be defined as plants which can complete their whole life cycle at salt concentrations higher than 200 mM NaCl [5,6]. Some of them can even withstand a long-term exposure to salt concentrations approaching those in salt water (*ca*. 600 mM NaCl). Halophytes encompass phylogenetically diverse species, both dicots and monocots, belonging to various plant families. They include plants inhabiting basically two types of natural habitats: (1) habitats with high levels of brackish water in soil which frame coastal lines both in tropical (e.g., mangrove ecosystems) as well as in temperate regions; (2) arid and semi-arid inland regions where annual evaporation rates exceed precipitation. Thus, salts are released from basal rocks, rise by capillary action to the upper layer of soil where they precipitate and cause soil salinization. The possibilities of utilization of halophytic plant species as agricultural crops are very limited although some exceptions, e.g., *Chenopodium quinoa* from Chenopodiaceae family or wild relatives of cultivated Triticeae such as tall wheatgrass (*Thinopyrum elongatum*), do exist [6,7].

Plants actively respond to stress by reprogramming their whole metabolism from growth and development to induction of an enhanced stress tolerance. Plant responses to salinity and mechanisms conferring plant salinity tolerance have been studied for a long time (reviews [2,3,8–10]). Recently, substantial progress in elucidation of salt tolerance mechanisms, especially salt ion signalling and transport, has been achieved due to utilization of modern genetic approaches (whole genome sequencing, reverse genetics methods, identification and characterization of key genes involved in salt stress signalling using *Arabidopsis thaliana* mutants; reviewed in [1,8,11,12]) and due to high-throughput methods of functional genomics (transcriptomics, proteomics, metabolomics, antioxidomics, *etc.*). In addition, proteins play an imminent role in plant stress response since they are directly involved in the acquisition of an enhanced stress tolerance. Therefore, proteomics of plant abiotic stress response has an immense potential in determining the key processes involved in plant stress response and stress tolerance acquisition (reviewed in [4]). Recently, a review focused on salinity response in important crop plants [13] and a comprehensive review on plant protein response to salinity including a database of salt-responsive proteins [14] have been published.

The aim of this review lies in an attempt to summarise recent knowledge on salinity response mechanisms in plants gained by utilization of high throughput “omics” methods. The attention is paid to the physiological aspects of plant response to salinity including proteins involved in signalling, changes in gene expression and protein metabolism and the resulting changes in cellular compartment composition and metabolism. Two major physiological effects of salinity—the immediate osmotic effect and the cumulative ionic effect—are discussed with respect to plant response at proteome level.

The results of proteomic studies carried out on a wide range of agricultural crops as well as wild plants including halophytic species are summarised with respect to the individual protein functional groups. The results achieved by proteomic studies are discussed in broader aspects of “omics” as well as physiological studies and differences underlying differential salt tolerance in glycophytes and halophytes are compared. The analysis of plant samples using “omics” methods results in production of large amount of data; however, the data itself have only limited value since data interpretation is important. For example, comparison of a salinity response in glycophytes and halophytes requires a use of related plant species, utilization of the same analysis methods, comparison and interpretation of the obtained data. The employment of *Thellungiella salsuginea*, an *Arabidopsis* salt-tolerant relative whose transcriptome can be studied using *A. thaliana* microarray has turned out a suitable model system for comparison of salt response in glycophytes and halophytes [15]. In case of rice, a halophytic wild rice species *Porteresia coarctata* seems to represent a promising model plant for comparative studies although cross-species matching of 2DE gels appears to be difficult due to quite large protein spot variation [16,17]. Comparison of related plant species with contrasting salinity tolerance could help us to unravel the molecular mechanisms underlying differential response of glycophytic and halophytic plants to the same environmental cues and to identify key genes and their transcript and protein products (including posttranslational modifications) responsible for an enhanced salinity tolerance in halophytes. Utilization of this knowledge in genetic engineering could lead to the improvement of salinity tolerance in economically important salt-sensitive species, namely the major crops such as rice, common and durum wheat, barley, soybean or potato which generally display relatively poor salinity tolerance.

## 2. Plant Adaptation to Salinity—The Role of Proteomics Regarding the Other Aspects of Plant Salinity Response

Plants reveal significant differences in their abilities to cope with salinity. It has been proposed by Inan *et al.*[15] that generally, three major factors could determine the tolerance of plants to extreme environmental conditions (abiotic stresses) at molecular level:

(1) Genomic level: Tolerant plants may possess some unique stress-responsive genes which are absent in susceptible plants (differences at genome structure level).
(2) Transcriptomic level: Tolerant plants reveal altered regulation of gene expression of important stress-responsive genes than susceptible plants (qualitative and quantitative differences at gene expression level);
(3) Proteomic level: Proteins involved in stress response reveal an altered activity in tolerant plants than in susceptible ones (differences in protein structure and activity level).

To the molecular levels listed above, metabolomic and physiological (functional) levels can be added (Table 1).

The salinity induces signalling events leading to changes in gene expression, protein relative abundance and activity. Proteins in turn induce profound changes in energy metabolism resulting in both short-term (stress response aimed at attenuation of direct impacts of stress such as changes in osmotic potential and salt ion activity) and long-term stress adaptations (structural adaptations of plant cells resulting in changes in plant growth and development). In accordance with aim of the review, the proteomic level of study will be emphasized below. However, physiological, genomic and transcriptomic levels will be listed briefly.

## 3. Effects of Salinity Stress on Plants (Physiological Level)

**Figure 1:**
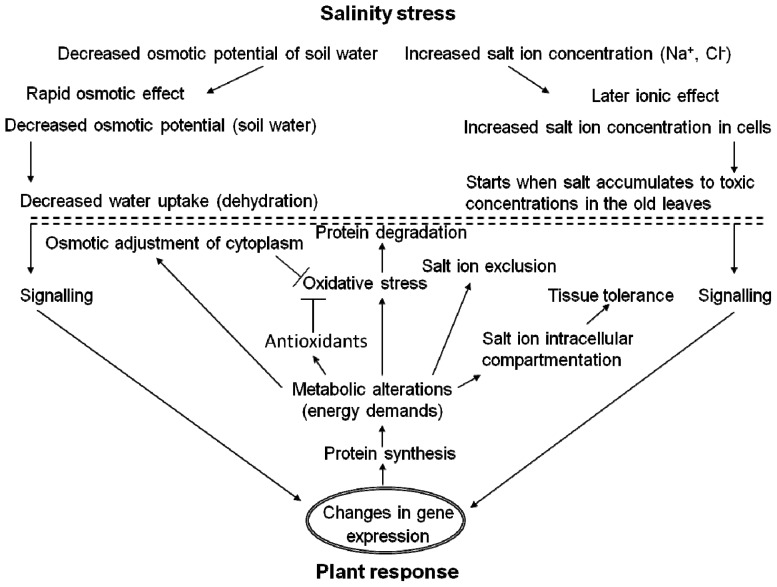
A schematic view on salinity effects on plants (osmotic effect and ionic effect) and a general plant response to salinity effects leading to signalling, changes in gene expression, changes in protein expression, changes in plant metabolism and effector responses leading to alleviation of adverse effects of the stress (osmolyte biosynthesis resulting in osmolyte adjustment—alleviation of osmotic stress; activation of ion (Na^+^) transport resulting in ion exclusion or intracellular (vacuolar) compartmentation—alleviation of ionic stress; alleviation of oxidative stress arising as a secondary stress due to imbalances in energy metabolism—activation of enzymatic and non-enzymatic antioxidant systems).

Generally, the plant response to salinity is dynamic revealing the same phases—an alarm phase, an acclimation phase, a resistance phase and an exhaustion phase—as described for a general plant stress response [4,36,37]. However, two major effects of salinity on plants could be distinguished [3]: a rapid, salinity non-specific osmotic effect, and a delayed, salinity specific ionic effect (Figure 1). Osmotic effect is caused by a decrease in osmotic potential of ambient soil water and it reveals a direct impact on plant root cell water status while ionic effect is caused by an increased accumulation of salt ions in plant cells to toxic levels; thus, it reveals a continuous, long-term effect of a cumulative nature. Both osmotic and ionic effects are reflected by a two-phase plant growth response, *i.e.*, a rapid and a delayed growth reduction response can be distinguished in salt-treated plants [1]. In salt-tolerant plants (halophytes), there are efficient mechanisms that could eliminate accumulation of salt ions in plant cells to toxic levels (ionic effect).

### 3.1. Osmotic Effect

An osmotic effect lies in a decrease of osmotic potential of soil water surrounding plant root cells due to dissolved salt ions. The osmotic effect reveals rapid, direct effects on plant root cells as a consequence of the difference between osmotic potential of cell cytoplasm and the surrounding soil water solution. Osmotic stress decreases water availability for the cells and it leads to a decreased water uptake resulting in cellular dehydration. It is a non-specific effect common to all dehydrative stresses (e.g., salinity, drought, low temperatures and some types of mechanical wounding). Under salinity conditions, an increased activity of salt ions results in a decreased osmotic potential as well as a passive salt ion penetration into plant cells. Both the decreased osmotic potential and the increased salt ion activity induce specific signalling events in plants.

#### 3.1.1. Osmotic Stress-Related Signalling

As a result of cellular dehydration, plant cell turgor decreases. A decrease in cellular turgor represents probably a signal of osmotic stress which is sensed at the cell plasma membrane. In *Arabidopsis thaliana*, a two-component histidine kinase AtHK1 could be considered a potential candidate for an osmosensor since it could complement a yeast osmosensing mutant [38]. Osmotic signal is then transduced via a series of phosphorylation (MAP kinase cascade) and calcium signalling to nucleus where it induces changes in gene expression. Besides phosphorylation cascades (MAP kinases MAPKKK, MAPKK, MAPK), osmotic stress signalling also involves plasma membrane phospholipid signalling (phospholipases C and D (PLC and PLD)) leading to formation of small signalling molecules inositol-1,4,5-trisphosphate (IP_3_), diacylglycerol (DAG) and phosphatidic acid (PA), respectively. These molecules induce Ca^2+^ signalling events leading to signal transduction to nucleus. It has also been found out that PLD activity in stomatal guard cells is coordinated with ABA and it seems very likely that PA mimics the effect of ABA in inducing stomatal closure (reviewed in [11]). Another important component of osmotic stress signalling is ABA function. Osmotic stress induces an enhanced ABA biosynthesis since an increased accumulation of the key ABA biosynthesis enzyme NCED (9-cis-epoxy-carotenoid dioxygenase) and an enhanced ABA accumulation have been observed [19]. ABA reveals a dual role in plant osmotic stress response. First, a rapid ABA response to osmotic stress directed by a decreased pH lies in induction of turgor loss in stomatal guard cells leading to stomatal closure. Second, a relatively delayed ABA response to osmotic stress lies in induction of several ABA-responsive transcription factors (so-called “*early response genes*”). These transcription factors then bind to ABA-responsive promoter elements (ABRE) in the promoters of several genes (so-called “*delayed response genes*”; e.g., *Lea/Rab* genes) which accumulate in plant cells to high levels and confer an enhanced stress tolerance [12].

ABA signalling also involves Ca^2+^ signalling as second messengers [39]. Potential ABA receptors include PYR/PYL/RCAR proteins which contain a so-called START (steroidogenic acute regulatory— StAR—related lipid transfer) domain capable of binding small hydrophobic molecules [40,41]. According to a model proposed by Klingler *et al.*[42], ABA binding to PYR/PYL/RCAR receptor leads to an interaction of the receptor with protein phosphatase 2C (PP2C). PP2C bound to PYR/PYL/RCAR receptor cannot inhibit the activity of SnRK2 kinase which is involved in phosphorylation of ABF2 factor. Phosphorylated ABF2 can bind to ABRE binding factor in the promoter of several ABA-responsive genes resulting in an up-regulation of their expression. Other possible ABA receptors are membrane-localized GPCR- type G proteins GTG1 and GTG2 [43]. ABA-dependent signalling pathways include AREBs (ABFs), bZIP transcription factors which bind to ABRE promoter elements in promoter regions of several ABA-inducible genes (*Cor*/*Lea*/*Rab*), DREB2A, DREB2B and CBF4/DREB1D TFs with an AP2 DNA-binding domain, NAC, MYB and MYC transcription factors (NACR, MYBR and MYCR promoter elements, respectively). In *A. thaliana*, it has become evident that AtMYC2 factor is also regulated by jasmonic acid. Thus, AtMYC2 functions in a crosstalk between ABA and jasmonic acid signalling [12].

Plant response to osmotic effect of salinity lies in osmotic adjustment. An enhanced accumulation of several osmotically active compounds results in a decrease of a cellular osmotic potential thus diminishing the difference between a cellular and an ambient osmotic potential. The cheapest, but quite harmful way to decrease the intracellular osmotic potential under salinity stress lies in an accumulation of salt ions in the cells. However, an enhanced accumulation of salt ions in cell cytoplasm causes an inactivation and degradation of the majority of cytoplasmic enzymes. Therefore, plants usually induce biosynthesis of several low-molecular, highly hydrophilic organic compounds as well as high-molecular hydrophilic proteins (e.g., LEA proteins) which do not only decrease intracellular osmotic potential, but also enhance protective properties on other cellular compounds adversely affected by dehydration [44]. Organic low-molecular-weight osmolytes include nitrogen-containing compounds such as free amino acids (proline, glutamic acid), quaternary ammonium compounds called betaines (glycine betaine GB) and polyamines (spermine, spermidine, putrescine); osmolytes without nitrogen include straight-chain polyhydric alcohols (glycerol, mannitol, sorbitol), cyclic polyhydric alcohols (D-ononitol, *myo*-inositol) and sugars (monosaccharides glucose, fructose, oligosaccharides sucrose, trehalose, raffinose-family oligosaccharides raffinose, stachyose and verbascose; reviewed in [1]).

### 3.2. Ionic Effect

At high salt levels, Na^+^ exclusion mechanisms are not sufficient for maintenance of low intracellular Na^+^ levels and Na^+^ ions start accumulating in plant tissues. In order to diminish their harmful effects on plant metabolic processes, they have to be compartmentalized internally via vacuolar sequestration. The major tonoplast proteins ensuring Na^+^ vacuolar sequestration belong to vacuolar H^+^-ATPases (V-ATPases) and H^+^-pyrophosphatases (H^+^-PPases) creating a sufficient electrochemical potential for energizing tonoplast membrane and NHX Na^+^/H^+^ exchangers which provide Na^+^ influx into vacuole in exchange with H^+^. Halophytes can be usually characterised by efficient compartmentation mechanisms underlying high tissue tolerance while glycophytes usually fail in establishing high tissue tolerance under long-lasting high salinity conditions [1,2].

#### 3.2.1. Ion-Related Signalling

Salinity stress is characterised by an increased salt ion activities in soil water surrounding plant root cells. As a consequence of activity gradients, salt ions, namely Na^+^, enter passively plant cell cytoplasm via non-selective cation channels because of the negative electrical potential (−120 mV) of plant cell cytoplasm. In addition, Na^+^ influx in cell cytoplasm is mediated by HKT (high-affinity potassium transporters) together with K^+^ ions. Ionic effect is a continuous, long-term effect of a cumulative nature since it is dependent on the intracellular salt ion levels which increase with the duration of salinity stress and with ageing processes of a given plant tissue.

It is generally assumed that an increased intracellular Na^+^ level (activity) induces Ca^2+^ signalling leading to an activation of Na^+^ active efflux from plant cells via SOS1/SOS2/SOS3 pathway recently characterised in *A. thaliana*[45,46]. First, Na^+^ induced Ca^2+^ signalling activates SOS3 which is a calcineurin B-type like calcium binding protein with a myristoyl anchor at the *N*-terminus. SOS3 with bound calcium can attach plasma membrane and interact with SOS2 which is a serine/threonine protein kinase. SOS3/SOS2 complex then activates SOS1 which is an ATP-dependent Na^+^/H^+^ exchanger with 12 transmembrane helices and a long intracellular chain which can be phosphorylated by SOS2/SOS3 complex. Active SOS1 ensures Na^+^ exclusion from plant cells via a mechanism of ion exchange (Na^+^ is exchanged by H^+^) at the cost of ATP. SOS1 also seems to control Na^+^ loading into xylem thus regulating Na^+^ level in transpiration stream and its root-to-shoot transport [47]. In addition to *SOS1*, *SOS2* and *SOS3* genes, *SOS4* gene has been identified in *A. thaliana* which encodes a pyridoxal kinase and its activity affects root hair development [48].

### 3.3. Further Aspects of Salinity Stress

Plant response to both osmotic and ionic effects of salinity stress puts enhanced demands on energy resources and energy metabolism. However, production of energy-rich compounds is usually severely reduced in most salt-sensitive plants due to an inhibition of CO_2_ assimilation which is a consequence of a decreased stomatal guard cell turgor and stomatal closure. Therefore, catabolic processes are being activated. Addition of soluble salts into nutrient solution induces an increase in plant respiration, a so-called “salt respiration” [49]. Nevertheless, some young tissues can even suffer from a lack of O_2_ available for respiratory processes due to stomata closure, and, as a result, activation of anaerobic processes such as glycolysis and ethanolic fermentation has been observed in some salt-sensitive plants. As a consequence of profound changes in energy metabolism, an enhanced risk of oxidative damage arises upon salinity stress. Oxidative stress is associated with an activation of several enzymatic and non-enzymatic ROS scavenging mechanisms, but also several defence- and pathogenesis-related proteins. In addition, the difference in osmotic potential between cell cytoplasm and ambient soil water and cell turgor loss poses a significant mechanical stress on plant cells leading to a profound reorganization of cell cytoskeleton and cell wall architecture. Therefore, it can be summed up that salinity stress affects a wide array of protein functional groups in plant cells. The major functional groups of proteins involved in salinity response and acquisition of salinity tolerance are discussed in the following sections.

## 4. Genomic Level of Adaptation to Salinity

Recent sequenation of the whole genome of *Thellungiella parvula* has revealed that although the genomes of *A. thaliana* and *T. parvula* are of very similar size (140 Mb) and gene number, there are significant differences in gene copy number in certain functional categories important for stress tolerance. *Thellungiella parvula* genome reveals a higher gene copy number of several genes involved in transport (ATPases *AVP1*, ion transporters *HKT1*, *NHX8*, ABC transporters) than *A. thaliana* genome while in contrast, *T. parvula* genome contains lower gene copy numbers of several genes involved in signal transduction with respect to *A. thaliana*[18].

## 5. Transcriptomic Level of Adaptation to Salinity—Comparative Studies

Due to proteomic focus of the review, only comparative transcriptomic studies will be listed in this subchapter. Comparative studies revealing differential gene expression have been carried out on related plant species with contrasting salinity tolerance such as *Arabidopsis thaliana* and *Thellungiella salsuginea* and rice and its wild relative *Porteresia coarctata* (Supplementary Table).

Comparative analysis of *A. thaliana* and *T. salsuginea* transcriptome under controlled conditions using *A. thaliana* microarray has revealed a constitutively increased relative abundance of several abiotic and biotic stress responsive genes such as superoxide dismutase (SOD), putative chitinase, antifugal protein-like PDF1.2, putative Δ-1-pyrroline-5-carboxylase (P5CS; an important enzyme involved in proline biosynthesis), β-glucosidase, Na^+^/H^+^ antiporter SOS1, cytochrome P450 like protein, putative vacuolar proton ATPase subunit, P-protein, peroxidase, plasma membrane proton ATPase, heat shock like protein Hsc70-3, several ABA-biosynthesis and ABA-responsive genes, and cell-wall biosynthesis related genes [19–21]. In contrast, the level of proline dehydrogenase (PDH) involved in proline catabolism was constitutively decreased in *T. salsuginea* than in *A. thaliana*[21]. A larger increased relative abundance of several enzymes involved in osmolyte biosynthesis (GS—glutamine synthase involved in proline biosynthesis, INPS—*myo*-inositol 1-phosphate synthase; galactinol synthase—galactinol is known to function as a precursor of raffinose-family oligosaccharides) has been detected in salt-treated *Thellungiella* than *Arabidopsis*. In contrast, a larger increased relative abundance of enzymes involved in jasmonic acid (JA) biosynthesis such as AOC (allene oxide cyclase) and LOX (lipoxygenase) has been found in *Arabidopsis* than in *Thellungiella*[22]. In *Arabidopsis*, a larger increased relative abundance of enzymes involved in energy production (glycolysis and respiratory pathway) was observed under salt stress while in *Thellungiella*, an increased relative abundance of photosynthesis related (RubisCO activase) and protein biosynthesis related (40S ribosomal proteins S7, S24, eukaryotic translation initiation factor 3A, ribosomal protein S15A) has been found in comparison to *Arabidopsis*. Therefore, it has been found out by a comparative analysis at both transcriptome and proteome levels [19,21,22] that salt stress causes larger changes in transcriptome and proteome of *A. thaliana* than *T. salsuginea* (e.g., 88 differentially abundant protein spots in *A. thaliana versus* 37 protein spots in *T. salsuginea* were found in plants grown under controlled condition; [22]). Therefore, salinity causes larger disturbances in the expression profile of *A. thaliana* cells than in the expression profile of *T. salsuginea* cells imposing a stronger stress on *A. thaliana. T. salsuginea* seems to be able to maintain a sufficient activity of photosynthetic apparatus and to produce enough amounts of ATP necessary for stress adjustment while *A. thaliana* cannot. These data point to the fact that susceptible plants can be characterised by mobilization of their energy reserves, consumption of energy reserves and an enhanced protein degradation under stress while tolerant plants are able to cope with the stress due to an enhancement of photosynthetic assimilation and protein biosynthesis as a result of their efficient protection. Thus, increased demands on energy and novel proteins as a consequence of an enhanced risk of protein damage can be satisfied in tolerant plants.

A transcriptomic study done by Kumari *et al.*[50] on a salt-sensitive rice line IR64 and a salt-tolerant rice Pokkali has led to identification of a set of salinity-responsive genes including GST, LEA, V-ATPase, OSAP1 zinc finger protein and transcription factor HB1B displaying a higher expression in Pokkali than in IR64 and possibly contributing to a higher salinity tolerance of Pokkali. Sengupta and Majumder [16] compared proteome of rice cultivars Pokkali, IR64 and halophytic wild rice (*Porteresia coarctata*) and revealed an enhanced abundance of proteins involved in protection and stabilization of both photosystems under stress (CP47 protein involved in stabilization of D1 protein in RC PSII, RC PSI subunit IV, 33 kDa Mn-stabilizing protein of OEC complex) as well as an increased abundance of RubisCO LSU and RubisCO activase. *Porteresia* revealed higher levels with respect to cultivated rice of several stress-protective proteins such as GS and *myo*-inositol-1-phosphate synthase involved in osmolyte biosynthesis and a dehydration-responsive CRT/DRE binding protein which belongs to transcription factors inducing expression of several LEA proteins. In wild rice, an enhanced abundance of cellulose synthase indicates an adaptation of cells on high osmotic pressure and on an enhanced risk of adverse structural changes in cell wall architecture under salinity condition. Finally, a preferred increased relative abundance of enzymes such as sucrose synthase (SUS) leading to production of UDP-glucose instead of invertase leading to glucose fits perfectly *Porteresia* strategy of energy saving under stress in order to use energy for an active stress acclimation.

## 6. Proteomic Level of Adaptation to Salinity

At proteomic level, plant response to salinity has been investigated in various plant species encompassing both salt-sensitive plants (glycophytes) and salt-tolerant plants (halophytes). In glycophytes, effects of salt stress have been studied in model plants such as *Arabidopsis thaliana*[51,52] and tobacco [53,54], dioecious poplar *Populus cathayana*[55], forage plants such as grasspea [56] and perennial *Poaceae* species creeping bentgrass *Agrostis stolonifera*[57], and practically all important agricultural crops such as rice [58–62], common and durum wheat [23,30,63,64], barley [65–69], foxtail millet [70], canola [71], sugar beet [24], soybean [72,73], pea [74], peanut [75], sorghum [76,77], maize [78,79], tomato [34,80], potato [81], cucumber [82], citrus [83] and grapevine ([25,84]; Supplementary Table S1A). Proteomics results of salt stress response in the major glycophytic crops (rice, wheat, barley, soybean, potato) have been summarised by Salekdeh and Komatsu [85] and Sobhanian *et al.*[13]. Considering halophytes, proteomic studies have already been published on arid plants *Suaeda aegyptiaca*[26] and *Suaeda salsa*[86], a leaf tonoplast fraction of *Mesembryanthemum crystallinum*[87], leaves of *Aster tripolium*[88], monocotyledonous Chinese halophyte *Puccinellia tenuiflora*[28], mangrove plant *Bruguiera gymnorhiza*[35], temperate coastal plant *Salicornia europaea*[27], *Thellungiella salsuginea*, a halophytic plant closely relative to *Arabidopsis thaliana*[21,22], a salt-tolerant model moss *Physcomitrella patens*[89], a halophytic alga *Dunaliella salina*[31,32] and a cyanobacterium *Synechocystis* PCC6803 ([90]; Supplementary Table S1B). Moreover, there have already been published a few studies comparing proteome response to salt stress in related plant species with contrasting salinity tolerance such as *Arabidopsis thaliana* and *Thellungiella salsuginea*[22], rice and salt-tolerant wild rice *Porteresia coarctata*[16], common wheat (*Triticum aestivum*) cv. Jinan 177 and *Triticum aestivum*/*Thinopyrum ponticum* introgression hybrid Shanrong 3 ([23,64]; Supplementary Table S2). The details on plant growth conditions, salinity treatments as well as the methods used for protein identification are given in Supplementary Tables S1 and S2. Densitometric analysis of 2DE gels coupled with protein identification by mass spectrometry has enabled the researchers to identify differentially abundant protein spots. However, a mere protein differential abundance does not give much information on protein function under salinity and therefore validation of comparative proteomics should be done by protein functional analysis. Therefore, other approaches (e.g., post-translational modifications (PTMs), protein-protein interactions, tissue and subcellular localization and phenotype influence on silencing or overexpressing of a gene encoding a protein of interest) have to be employed to unravel the role of the proteins in acquisition and development of salinity tolerance in plants.

Regarding plant physiological responses to salinity, the impact of proteins can be observed regarding responses to both osmotic and ionic effects of salinity. Considering the osmotic effect which causes not only a significant osmotic, but also mechanical stress on plant cells, an enhanced biosynthesis of several osmotically active organic compounds as well as proteins with osmoprotective functions such as LEA proteins could be mentioned. In addition, profound cytoskeleton reorganization affecting cell wall growth and development and an enhanced abundance of plasma membrane coat proteins forming a network of oligomers with protective mechanical function has been found in a halotolerant alga *Dunaliella salina*[32]. Regarding physiological mechanisms coping with adverse effects of salt ions, changes in protein relative abundance and activity seem to affect the efficiency of both salt ion exclusion and salt ion intracellular compartmentation mechanisms. Considering salt ion exclusion mechanisms, the proteomic study on a halophytic plant *Salicornia europaea* by Wang *et al.*[27] has clearly shown that the mechanism of salt ion exclusion via transpiration-mediated bypass flow employed by the plant is associated with an increased abundance of several plasma membrane-associated transmembrane ion transporters such as SOS1, NhaA (Na^+^/H^+^ antiporter), VDAC (voltage-dependent anion channel) and calcium-mediated sensors. The mechanism of cellular salt ion exclusion is coupled with a long-distance salt ion translocation via a transpiration-mediated bypass flow. Therefore, an increased lignification associated with an enhanced SAM biosynthesis (SAM is an important methylation agent involved in lignin biosynthesis) and a development of well-lignificated xylem vessels have been found out in *Salicornia*. Considering salt ion intracellular compartmentation, a development of large vacuoles in root cells of a tropical mangrove plant *Bruguiera gymnorhiza* coupled with an enhanced abundance of FBP aldolase has been observed by Tada and Kashimura [35]. FBP aldolase could play an important role in salt ion vacuolar compartmentation since this enzyme is known to directly physically interact with and activate tonoplast H^+^-ATPase by stimulating its ATP binding and ATP hydrolysing activity which is crucial for salt ion import into vacuole [87].

**Figure 2:**
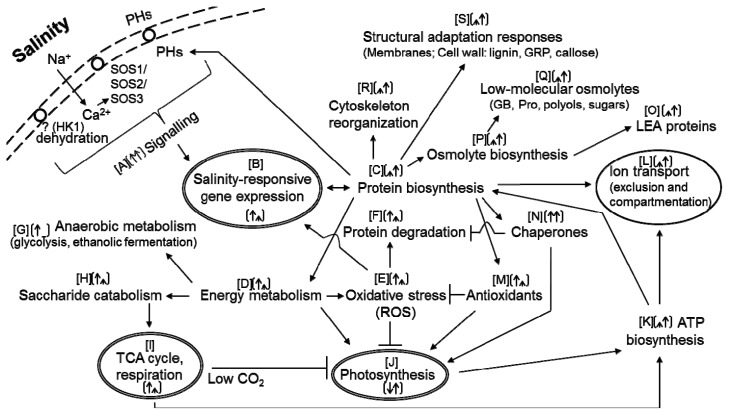
A summarising scheme showing general plant response to salinity at cellular level and indicating differences between glycophytes (left arrows in diagrams showing quantitative changes) and halophytes (right arrows in diagrams showing quantitative changes). Arrow (↑) means an increased relative abundance and arrow (↓) means a decreased relative abundance of a given plant response (metabolic process) under salinity, respectively, deduced on the results of comparative proteomic studies. The length of the arrow indicates the relative magnitude of a quantitative change deduced from changes in relative abundance of the proteins that participate in a given process (Table 2). Two lengths of arrows were used in the scheme; in case of short arrows, the quantitative change in protein abundance in proteins involved in a given process is generally smaller than in case of long arrows. References: (**A**) Signalling [19,21,22,89]; (**B**) Salinity-responsive gene expression [22,76,91]; (**C**) Protein biosynthesis [22,59,61]; (**D**) Energy metabolism [22,23,61]; (**E**) Oxidative stress [60,72,74]; (**F**) Protein degradation [26,60,70,78]; (**G**) Anaerobic metabolism [56,58,73]; (**H**,**I**) Saccharide catabolism; TCA cycle, respiration [22,28,62]; (**J**) Photosynthesis [16,26,29]; (**K**) ATP biosynthesis [22,61,70,86,88]; (**L**) Ion transport [22–24,51,57,82,87]; (**M**) Antioxidants [22,23,28,60,61,72,76,80,88,89]; (**N**) Chaperones [22,25,72,74,75,81,84,89,92]; (**O**) LEA proteins [33,92–95]; (**P**) Osmolyte biosynthesis [19,21–23,26]; (**Q**) Low-molecular osmolytes [19,21,22,26,70]; (**R**) Cytoskeleton reorganization [22,26,60,65,73,82]; (**S**) Structural adaptation responses [27,28,34,57,70,78].

Several functional groups of proteins affected by salt stress (summarised in Figure 2 and Table 2) include proteins involved in signalling, ion transport, energy metabolism (photosynthesis, ATP production, respiration), carbohydrate, protein and lipid metabolism, metabolism of osmolytes and phytohormones, stress-related proteins (oxidative stress—ROS scavenging enzymes, pathogenesis related proteins, osmotic stress related proteins—osmotin), cytoskeleton and cytoskeleton-associated proteins, enzymes involved in secondary metabolism (biosynthesis of lignin, degradation of cyanate) and others. Below, the main protein functional groups affected by salt stress are discussed in a greater detail.

### 6.1. Signalling

Most data on salinity (both osmotic effect and ionic effect) signalling pathways including the SOS1/SOS2/SOS3 signalling pathway in *A. thaliana* have been gained at genomic (utilization of mutant plants) and transcriptomic levels. In proteomic studies, there are only isolated reports on differential protein abundance of some salinity signalling involved proteins. The main reason of these results lies in a relatively low abundance of signalling proteins with respect to other cytosolic proteins making the signalling proteins hardly detectable on 2DE gels. In 2DE experiments, some Ca binding proteins activated by salinity have been detected including plasma membrane protein annexin [22] and calmodulin (CaM; [91]). Ca signalling transduces the salinity signal from plasma membrane to nucleus where profound changes in gene expression are induced. Other differentially abundant signalling proteins include subunits of plasma membrane associated heterotrimeric GTP binding proteins and small G proteins (Rab GTPases, especially Rab5 and Rab7 families) which are involved in stress signal transduction via regulation of endocytosis and vesicle trafficking [22,23,51,52,59]. Furthermore, an increase in mitogen-activated protein kinase MPK4, a negative regulator of SA signalling and systemic acquired resistance, and a positive regulator of JA signalling, was observed under salinity [22,96]. Induction of several other mitogen-activated serine/threonine protein kinases (MAPK) and lectin-like protein kinases has been found in grasspea [56] and sorghum [76]. In salt-treated rice root cells, a new salt-responding leucine-rich-repeat (LRR) type receptor-like protein kinase OsRPK1 was identified by Cheng *et al.*[91]. An increased relative abundance of 14-3-3 like proteins which are known to interact with MAPK kinase cascade, to regulate the activity of plasma membrane H^+^-ATPases and to contribute to a maintenance of intracellular ion homeostasis has been found in *Physcomitrella patens* gametophyte [89], common wheat Jinan 177 and *T. aestivum*/*Thinopyrum ponticum* introgression hybrid [23], in maize root [79] and grasspea leaves [56].

### 6.2. Gene Expression and Protein Metabolism

Changes in gene expression and an enhanced risk of protein damage induce profound alterations in DNA remodelling, transcription and protein metabolism—protein biosynthesis as well as protein degradation. In proteomic studies, increased relative abundance of several DNA remodelling enzymes such as DNA topoisomerase and DNA helicase has been found indicating an altered and enhanced gene expression activity. Salt-induced increased relative abundance of several transcription factors, namely from NAC family, was reported in salt-treated tomato [80], rice root [62] and barley leaf [65]. NAC-α proteins may be also involved in protein sorting and translocation of nascent (unfolded) polypeptides to endoplasmic reticulum. Increased relative abundance of nucleic acid binding protein observed in salt-exposed durum wheat is crucial for RNA posttranscriptional regulation and it seems important for acquisition of salt tolerance in durum wheat [30]. Consistently with profound changes in gene expression (DNA remodelling and transcription processes), changes in abundance of several proteins involved in protein biosynthesis have been detected. Increased relative abundance of several translation initiation and elongation factors (e.g., eukaryotic initiation factor eIF3A, eukaryotic elongation factors eEF1B alpha 2 subunit and eEF2 in salt-treated *A. thaliana*; [22]) and profound changes in abundance in several ribosomal proteins (40S ribosomal proteins S2, S7, S24, S29; 60S ribosomal proteins L5, L12, L13A, L29, L39) have been found [22,59,61,84].

An active stress acclimation and salinity tolerance acquisition processes are conferred by an enhanced biosynthesis of several novel proteins. In relatively salt-tolerant plants (both halophytes and glycophytes), an enhanced protein biosynthesis is reflected by an enhanced nitrogen assimilation and amino acid biosynthesis. Enhanced abundance of glutamate ammonia ligase which plays an important role in nitrogen assimilation and amino acid biosynthesis was found in *Sorghum bicolor*[76] and an enhanced abundance of several aminotransferases, namely glutamine synthetase (GS), was observed in salt-tolerant plants such as *Salicornia europaea*[27] and *Thellungiella salsuginea*[22], but also in rice roots [62]. Glutamic acid is not only a protein component, but also an important organic osmolyte. In contrast, in salt-sensitive plants such as potato, a decreased relative abundance of some proteins involved in protein and amino acid biosynthesis such as an mRNA binding protein and GS has been observed [81]. Similarly, in salt-treated *Arabidopsis* cell culture, a decreased relative abundance of several proteosynthesis-related proteins such as eukaryotic translation initiation factor eIF-4E2, putative elongation factor EF2 or tRNA synthase class II was observed [52] indicating a supression of protein biosynthesis upon salt stress. Considering protein degradation, increased relative abundance of proteasome subunits, e.g., proteasome subunit alpha type 6 in *Suaeda aegyptiaca*[26], putative alpha 1 subunit of 20S proteasome in rice panicles [60] or 26S proteasomal subunit in foxtail millet [70] and *Salicornia europaea*[27] indicates an enhanced protein degradation upon salt stress. Increased relative abundance of FtsH-like protein, an ATP-dependent metalloprotease involved in degradation of D1 core component of PSII, indicates an enhanced damage of photosystem core proteins upon salinity [78].

### 6.3. Energy Metabolism

Salinity stress reveals profound impacts on plant energy metabolism. Due to osmotic stress leading to stomata closure and a reduced CO_2_ availability, CO_2_ assimilation becomes decreased in most plants. A reduced CO_2_ assimilation rate is reflected by a decreased abundance of RubisCO large (RubisCO LSU) and small (RubisCO SSU) subunits, OEE proteins (components of OEC complex), carbonic anhydrase and RubisCO activase and an enhanced degradation of RubisCO subunits observed in several glycophytic plants and crops exposed to salt [22,56,71,73,81]. However, in some sensitive plants such as durum wheat, a salt-induced increased relative abundance of RubisCO activase has been observed probably as plant response to support RubisCO carboxylase activity [30]. Besides activation of RubisCO via removal of pentose phosphates thus enabling RubisCO carbamoylation, RubisCO activase reveals also a chaperone function under stress [61,65]. Similarly, an increased relative abundance of OEE2 protein was found in salt-treated barley [65,66], possibly as a compensation for stress-induced damage of PSII core. An enhanced abundance of ferredoxin NADPH reductase, 23 kDa polypeptide of PSII and the FtsH-like protein observed in salt-exposed maize chloroplast fraction may be associated with an attenuation of adverse impacts of Na^+^ on photosynthetic electron transport chain [78]. Other salt-increased proteins with protective functions on photosystems include CP47 protein revealing protective effects on D1 protein in RC PSII and PSI subunit IV protein [16,70].

A reduced CO_2_ assimilation is reflected by a decreased abundance of several CO_2_ assimilation related enzymes in plants. A decreased abundance of phosphoribulokinase (PRK) and phosphoglycerate kinase (PGK) was found in durum wheat [30] and canola [71] under salinity. PRK catalyses an ATP-dependent phosphorylation of ribulose-5-phosphate to ribulose-1,5-bisphosphate, a key step in pentose phosphate pathway (PPP) necessary for CO_2_ assimilation. PGK utilizes ATP to phosphorylate 3-phosphoglycerate to form 1,3-bisphosphoglycerate; the reaction represents the first reaction in the reduction step of Calvin cycle. A decreased abundance of transketolase (TK) has been observed in salt-treated rice [61]. TK is known to catalyze a transfer of a two-carbon residue from fructose-6-phosphate to glyceraldehyde-3-phosphate leading to a regeneration of ribulose-1,5-bisphosphate (RuBP), a substrate for RubisCO. A decreased abundance of TK thus indicates a decreased regeneration of RuBP and a decreased RubisCO carboxylase activity. In contrast, other enzymes associated with carbohydrate metabolism (Calvin cycle, glycolysis) have been found increased under salt stress. As an example, fructose-1,6-bisphosphatase (FBPase) and fructose-1,6-bisphosphatase aldolase (FBP aldolase) can be given [61]. FBPase catalyses hydrolysis of fructose-1,6-bisphosphate to fructose-6-phosphate and FBP aldolase catalyses conversion (splitting) of fructose-1,6-bisphosphate into glyceraldehyde-3-phosphate and dihydroxyacetone phosphate. Increased relative abundance of FBP aldolase together with other glycolytic enzymes and enzymes involved in ethanolic fermentation and glycolate metabolism in rice seedlings indicates an increased relative abundance of anaerobic metabolism under salt stress [58]. An increased relative abundance of alcohol dehydrogenase (ADH) has been found in hypocotyls of salt-stressed soybean [73] and grasspea leaves [56]. Moreover, it has been hypothesized that FBP aldolase could play a role in salt ion vacuolar compartmentation since it can directly physically interact with tonoplast H^+^-ATPase and activate its transport function [35,87].

It should also be taken into account that apart from cytosolic or chloroplastic isoforms involved in glycolysis or Calvin cycle reactions, several carbohydrate metabolism-related enzymes have also nuclear isoforms with important regulatory functions on gene expression and posttranscriptional regulation [97]. For example, nuclear isoform of glyceraldehyde-3-phosphate dehydrogenase (GAPDH) has been proposed a tRNA-binding protein participating in RNA export [98]. Nuclear isoform of FBP aldolase has been proposed a DNA-binding protein inducing the expression of its own gene [99]. Nuclear isoform of enolase has been described as *LOS2* gene in *A. thaliana* and has been shown to positively regulate the expression of *CBF* genes [100]. Therefore, it should always be considered that different subcellular isoforms of the same protein should reveal diverse biological functions.

It can thus be concluded that in glycophytes, photosynthesis (CO_2_ assimilation) is decreased by salt stress. However, in some halophytic plants, maintenance or even an increased net photosynthesis rate under salt stress has been found. A salt-induced increase in the level of RubisCO LSU, RubisCO SSU as well as LHC chlorophyll *a*/*b* binding protein was detected in *Physcomitrella patens* gametophyte [89] or in *Porteresia coarctata* leaves [16]. In halophytic plant *Suaeda aegyptiaca,* an increased abundance of D2 protein, a core protein of PSII which is severely damaged under stress conditions and causes an impairment of the whole RC PSII function has been found. In addition, an enhanced abundance of choline monooxygenase (CMO), a key enzyme in GB biosynthesis, has been found in salt-treated *S. aegyptiaca*. It has been hypothesized that an increased accumulation of GB contributes to stabilization of D2 protein and the whole RC PSII core complex thus contributing to maintenance of photosynthetic electron flow under stress. An enhanced accumulation of CP47 protein seems also to be associated with stabilization of D1 protein in salt-treated *Porteresia*[16]. Another strategy which seems to be suitable under conditions of restricted CO_2_ (stomata closure) and water availability lies in C4 photosynthesis. An increased abundance of NAD-malic enzyme has been detected in *Aeluropus lagopoides*, a halophytic C4 plant from *Poaceae* family, while a decrease in Calvin cycle enzymes was observed [29].

In response to salt stress, an increased relative abundance of several proteins involved in metabolic processes leading to energy production (energy release) such as glycolysis, tricarboxylic (TCA) acid cycle, photorespiratory pathway and pentose phosphate pathway (PPP) has been found. In a comparative proteomic study of salt-treated *Arabidopsis thaliana* and *Thellungiella salsuginea*, a higher relative abundance of respiration-related enzymes was found in *Arabidopsis* than in *Thellungiella*[22]. The increased enzymes in *A. thaliana* included carbonic anhydrase, glyceraldehyde-3-phosphate dehydrogenase, succinyl-CoA ligase β subunit, mitochondrial malate dehydrogenase (MDH-NAD), glucose-6-phosphate isomerase and pyruvate dehydrogenase [22,52]. Similarly, an increased relative abundance of cytochrome c oxidase subunit 6b-1 has been observed in salt-treated rice root [62].

Plant responses to salinity pose enhanced demands on energy production. Therefore, an enhanced abundance of several subunits of ATP synthase, namely subunit β, involved in ATP biosynthesis has been found in several tolerant salt-treated plants [22,61,70,86,88] while in some susceptible plants, a decreased relative abundance of ATP synthase subunits indicates an impairment in acclimation to salinity. Alterations in abundance of several subunits of CF1-CF0 complex of chloroplast ATP synthase have been observed in maize chloroplast fraction [78] and a decreased relative abundance of mitochondrial ATP synthase subunit β-3 has been found in cucumber roots [82]. In addition, an increased relative abundance of enzymes involved in ATP biosynthesis (adenylate kinase ADK) and energy salvage (nucleoside diphosphate kinase 1 (NDPK1) involved in interconversion between ATP and CTP, GTP and UTP) have been observed in salt-tolerant plants such as *Suaeda salsa*[86], but also in pea [74] and wheat [63]. In *Arabidopsis*, NDPK interacts with MAPK-mediated H_2_O_2_ signalling, downregulates the production of ROS and enhances stress tolerance [101].

### 6.4. Oxidative Stress and Stress-Related Proteins

Consistent with profound alterations in energy metabolism, there is an increased risk of oxidative damage in salt-treated plants. Similarly to metabolic changes, the risk of oxidative stress is generally higher in glycophytes than in halophytes. Enhanced ROS formation results in an increased relative abundance of several ROS scavenging enzymes such as CAT, SOD, enzymes of ascorbate-glutathione cycle APX, MDAR, DHAR [67,74,80], cytochrome P450 monooxygenase, thioredoxin *h* (Trx *h*), glutathione-*S*-transferase (GST), *etc.*, and other proteins involved in maintenance of plant redox status (protein disulfide isomerase; [22,60,61,76,81,88,89] and others). However, regarding various enzymatic isoforms of some ROS scavenging enzymes such as APX or MDAR, there has also been a decrease found under salinity indicating a complex manner of regulation [51]. Increased relative abundance of 2-Cys peroxiredoxin (PRX), an enzyme involved in H_2_O_2_ detoxification and energy dissipation in photosynthetic apparatus in salt-treated *Aeluropus lagopoides* is probably associated with an enhanced C4 photosynthesis under salinity [29].

Besides activation of ROS scavenging enzymes, other responses could be observed in salt-treated plants. An increased abundance of tocopherol cyclase, a crucial enzyme in the biosynthesis of α-tocopherol, an important compound in plant non-enzymatic ROS scavenging mechanisms, has been found in Chinese halophytic plant *Puccinellia tenuiflora*[28]. An increased abundance of mitochondrial alternative oxidase (AOX), an enzyme involved in so-called “cyanide-resistant respiration,” *i.e.*, respiration without utilization of cytochromes leading to a reduced ROS formation, has been observed in salt-treated wheat mitochondria [63]. It is also known that free metal ions could act as catalysers of ROS formation. Therefore, responses leading to elimination of free metal ions were observed in salt-stressed plants. In salt-exposed barley roots, a decrease in IDI2, IDS2 and IDS3 proteins has been observed [68]. IDI2 encodes α subunit of eIF2B regulating protein synthesis in stress adaptation processes and IDS2 and IDS3 proteins catalyze condensation of 2′-deoxymugineic acid to form mugineic acid that belongs into phytosiderophores [68]. Phytosiderophores are known as Fe chelators; therefore, a decreased Fe uptake from ambient soil could be expected under salt stress conditions. The reduced relative abundance of IDI2, IDS2 and IDS3 indicates a decrease in Fe consumption which may reflect the avoidance of metal ion-induced oxidative stress. Consistent with the aim to maintain low levels of free intracellular Fe, an increased abundance of ferritin, an Fe-binding protein, was found in salt-treated rice [61], *Arabidopsis* cell culture [52] and tomato [80] seedlings, and accumulation of triplicated transferrin-like protein was detected in *Dunaliella salina*[31]. Induction of Osr40c1 protein with a histidine-rich metal-binding domain has been found in plasma membrane fraction of young rice roots under salinity [91] and an increased relative abundance of metallothionein, a protein involved in binding metals, but also in ROS detoxification, has been found in foxtail millet [70]. An enhanced risk of oxidative stress under salinity is also reflected by an increased abundance of protoporhyrinogen IX oxidase, the final enzyme of heme and chlorophyll biosynthetic pathway, which was found in salt-treated maize chloroplasts [78] since protoporhyrinogen can be involved in singlet oxygen formation. Similarly, an increased level of FutA1 and FutA2 which are an Fe-binding lipoprotein and a periplasmic Fe-binding protein, respectively, in plasma membrane fraction of salt-treated *Synechocystis* indicates a strong interrrelation between salinity and Fe-deficiency stress in cyanobacteria [90]. An elevated abundance of magnesium chelatase, an enzyme involved in incorporating magnesium into chlorophyll structure which catalyses the first unique step of the chlorophyll biosynthetic pathway, has been found in salt-treated barley [66].

Salt stress also causes an augmentation of several proteins with protective functions such as chaperones from HSP90 family in tomato roots [34] and HSP70 family, Hsc70 (heat-shock cognate) proteins in *A. thaliana*[22] and *P. patens*[89], DnaK protein, and others in salt-treated rice seedlings [59,61] or *Arabidopsis* cell culture [52]. Increased relative abundance of several small HSPs (mitochondrial small HSP, chloroplast HSP, 17.8 kDa class I small HSP, HSP20) was found in salt-treated tomato hypocotyls [80] and *Aster tripolium* leaves [88]. Increased relative abundance of STI1 protein, a stress-responsive protein with two heat shock chaperonin-binding motifs and three tetratricopeptide repeats (TPR) in salt-treated rice panicles [60] points toward a large regulatory network affected by salt stress since TPR-containing proteins have been reported as being involved in myriads of processes including HSP90 signalling, gibberellin signalling and protein mitochondrial transport. Several pathogenesis-related proteins such as PR5 protein, PR10 proteins, TSI-1 protein, elicitor peptide three precursor and β-glucosidases have been found as being induced by salinity [22,25,30,67,72,75,81,84]. PR10 proteins have been reported as being involved not only in defence reactions, but also in JA signalling [102], they could reveal ribonuclease activity [103] and could bind several ligands including cytokinins, fatty acids, flavonoids and brassinosteroids [104]. β-glucosidases catalyse hydrolysis of 1,3-β-d-glucosidic linkages in 1,3-β-d-glucans and they are implicated in three processes: (1) alterations of specific β-linked polysaccharides during cell expansion in development; (2) pathogen defence response by cyanogenesis since the enzymes catalyse hydrolysis of cyanogenic glucosides after pathogen attack; (3) regulation of phytohormone activity via the release of active cytokinins, gibberellins and auxins from biologically inactive hormone-glucoside conjugates.

Other stress-responsive proteins include germin like proteins (GLP) which play an important role in plant embryogenesis. Some germin-like proteins display oxalate oxidase and SOD activities. Increased relative abundance of germin-like proteins was observed under several abiotic and biotic stress conditions, for example in salt-stressed barley leaves [65] and *Arabidopsis* roots [51]. Another interesting protein group induced by salinity are lectins which are known as being involved in protein-saccharide interactions and stress signalling. Small lectins with a jacalin domain have been proposed to function in plant defence mechanisms [105]. An increased relative abundance of a jacalin lectin family protein was observed in salt-treated *A. thaliana* leaves [22] and an induction of mannose-binding RICE lectin (MRL) was reported in rice roots after a short-term salt stress [59] while a decreased relative abundance of lectin-like protein was found in salt-treated soybean root and hypocotyl [72].

Other stress-protective proteins, such as osmotin and osmotin-like proteins are associated with a plant adjustment to an enhanced osmotic stress. Increase in osmotin and osmotin-like proteins has been found in various salt-treated plants ranging from a salt-tolerant cultivar of potato [81] and hypocotyls of tomato [80] to roots of a halophytic mangrove plant *Bruguiera gymnorhiza*[35].

### 6.5. Osmolyte Metabolism

A decreased osmotic potential of water containing high concentrations of dissolved salt ions poses an enhanced osmotic stress on plant cells, especially root cells which are in a direct contact with soil water. The response of plant cells to a decreased ambient soil osmotic potential lies in a decrease of intracellular osmotic potential which represents the principle of osmotic adjustment. The mechanisms of osmotic adjustment play an important role in prevention of plant cell dehydration, loss of turgor, and finally, plant cell plasmolysis (plasma membrane detached from cell wall, attached only via plasmodesmata—the phenomenon observed in root cells which are in contact with ambient soil water) or cytorrhesis (the volume of the cells shrinks, but plasma membrane remains attached to the cell wall—the phenomenon found in shoot cells which are surrounded by air, not by soil water [106]). These changes reveal deleterious effects on plant plasma membrane as well as cytosolic structure and function (plasma membrane signalling, transport, *etc*.; reviewed in [2]).

Basically, plant cells can achieve osmotic adjustment by an accumulation of inorganic salt ions which represents a cheap, low energy-cost way; however, a harmful way due to an inhibition of many intracellular enzymes by high salt concentrations. The other way lies in an accumulation of organic hydrophilic compounds, so-called osmolytes. In salt-treated plants, changes in metabolism of several osmolytes, namely proline and GB, have been found. An increased level of glutamine synthase (GS) and Δ-pyrroline-5-carboxylate synthase (P5CS), enzymes involved in proline biosynthesis, and in contrast, a decreased level of proline dehydrogenase (PDH), an enzyme catalysing proline hydrolysis, have been found in *Thellungiella* not only under salt stress, but even under non-stress (control) conditions [19,21,22]. An increased relative abundance of enzymes involved in GB biosynthesis such as SAM synthase (SAMS), choline monooxygenase (CMO) and betaine aldehyde dehydrogenase (ALDH) has been found in salt-treated *Suaeda aegyptiaca*[26] and foxtail millet [70]. Increased relative abundance of l-*myo*-inositol-1-phosphate synthase (INPS) was found in salt-treated rice relative *Porteresia coarctata*[16]. In addition to low molecular osmolytes, an increased biosynthesis of several hydrophilic proteins with osmoprotective function (e.g., proteins from LEA superfamily including dehydrins) has been found (reviewed in [44,107,108]). As examples of salt-inducible LEA proteins, dehydrin TAS14 in tomato [93], several dehydrins and LEA3 proteins in salt-tolerant Indica rice cultivars Pokkali and Nona Bokra [94], phosphorylated DHN5 in durum wheat [95], PeuDHN1 in poplar *Populus euramericana*[92] and AmDHN1 in gray mangrove *Avicennia marina*[33] can be given. Plants thus solve the problem of a decrease in ambient osmotic potential by accumulation of newly synthesized organic compounds; in contrast, a tight regulation of inorganic salt ions occurs in salt-treated plants.

### 6.6. Ion Transport

Control of salt ion transport is a crucial feature for achievement of plant salt tolerance since intracellular enzymes are inhibited by elevated salt ion concentrations both in glycophytes and halophytes. Therefore, maintenance of low levels of salt ions is pivotal for all plants. Plant strategies show that achievement of low intracellular salt ion concentrations can lie either in salt ion exclusion from cytoplasm into apoplast or intracellular salt ion compartmentation, leading mostly to salt ion accumulation in vacuole.

Generally, salt ions (mostly Na^+^) enter plant cells via plasma membrane non-selective cation channels and also via HKT (high potassium affinity transporters). Salt ion exclusion into apoplast is conferred by the activity of several plasma membrane located ATP-dependent transmembrane ion channels, utilizing mostly Na^+^/H^+^ antiport which does not result in changes in electrical charge. Probably the best characterised plasma membrane Na^+^/H^+^ antiporter is SOS1 protein in *A. thaliana*[46]. The activity of Na^+^/H^+^ antiporters is supported by of plasma membrane H^+^-ATPases creating a sufficient H^+^ gradient. A constitutively enhanced abundance of plasma membrane H^+^-ATPases has been found in relatively salt-tolerant sugar beet [24].

Vacuolar Na^+^ compartmentation is conferred by the activity of several tonoplast Na^+^/H^+^-antiporters from NHX family whose ion exchange activity is supported by H^+^-ATPases, so-called V-ATPases, and H^+^-pyrophosphatases (H^+^-PPases) whose abundance increases upon salt stress [22,23,51,57,82]. It has been shown by Barkla *et al.*[87] that tonoplast V-ATPases, especially their VHA-B subunit, interact with glycolytic enzymes, namely aldolase and enolase, which seem to stimulate V-ATPase hydrolytic activity. It has also been shown by Batelli *et al.*[109] that the activity of V-ATPases can be stimulated by SOS2.

### 6.7. Mechanical Stress-Related Proteins

Besides cellular dehydration and osmotic adjustment, a decrease in ambient osmotic potential poses also a substantial mechanical stress on plant cells as a consequence of changes in cytoplasmic volume. Therefore, changes in abundance of several cytoskeletal and cytoskeleton-associated proteins (actin, tubulin; profilin—an actin-binding protein involved in polymerization and depolymerization of actin filaments; kinesin—a microtubule motor involved in microtubule-dependent transport processes, especially during cell cycle and cytokinesis) have been observed in salt treated plant cells [22,26,27,60,65,73,82]. An increased relative abundance of a protein related to heavy-chain plant-specific myosin VIII was found in plasma membrane fraction of salt-stressed rice roots [91]. This protein has been proposed to act as a cytoskeleton-cell wall linker and it is known to bind to callose synthase complexes in plasma membrane whose activation is a part of a plant stress response. Similarly, an increased relative abundance of remorin, a plant-specific plasma membrane/lipid raft-associated filamentous protein indicates a profound reorganization of membrane skeletons, structures involved in plasma membrane integrity and in organization of membrane domains harboring signalling complexes and ion channels [91]. An increased relative abundance of cellulose synthase in salt-treated *Porteresia* indicates a need to adapt to an enhanced osmotic pressure of the ambient environment. Moreover, it should be a response on increased Na^+^ levels in cell wall and an enhanced risk of replacement of cell wall Ca^2+^ by Na^+^[16]. An increased level of β-d-glucan exohydrolase found in creeping bentgrass can be associated with increased cell wall plasticity in response to salt stress [57]. In contrast, a decreased relative abundance of xyloglucan endotransglycosylase (XET), an enzyme involved in cell wall elongation, has been observed in grapevine cultivar Chardonnay during a 16-d salt treatment which is consistent with the generally observed reduction of growth [84]. An increased relative abundance of cell-wall associated glycine-rich proteins (GRP) revealing both mechanical and defence properties has been observed in rice panicles [60] and cucumber seedling roots [82]. Changes in cytoskeletal as well as plasma-membrane associated proteins with mechanical functions indicate profound alterations in both intracellular and cell wall architecture of plant cells facing the impacts of an osmotic stress.

Most plant cells contain cell wall which provides a mechanical protection. However, a halotolerant alga *Dunaliella salina* lacks a rigid cell wall; thus, an accumulation and oligomerization of several coat proteins with a mechanical function has been observed in its plasma membrane under high salt (3 M NaCl) levels [32].

### 6.8. Phytohormone Metabolism

Changes in abundance of several enzymes involved in phytohormone metabolism such as jasmonic acid (JA) biosynthesis (allene oxide cyclase AOC; lipoxygenase LOX), gibberellin (GA) biosynthesis (DWARF3), ethylene biosynthesis (SAMS) and ABA biosynthesis (NCED) have been detected in salt-treated plants.

Increased relative abundance of ABA biosynthesis (increase in NCED level) found in *Thellungiella salsuginea*[19] corresponds with enhanced ABA levels observed in salt-treated plants and with an increased expression of several early (ABA-dependent transcription factors) and delayed (genes induced by ABA-dependent transcription factors, e.g., *Lea/Rab* genes) ABA-responsive genes. An enhanced induction of ethylene receptor ETR1 was found in common wheat cv. Jinan under salinity [64].

Activation of JA biosynthesis (increase in AOC and LOX levels) in salt-treated *A. thaliana* indicates an increased relative abundance of JA-induced signalling under salt stress [22,51].

Increased relative abundance of *DWARF3* gene involved in GA biosynthesis under salt stress in *T. aestivum/Thinopyrum ponticum* hybrid indicates that the hybrid can cope efficiently with stress since GAs are known to activate many vital processes thus revealing an important antistress and antisenescence activity [23].

### 6.9. Lipid Metabolism

Changes observed in lipid metabolism in salt-treated plants can be associated with adverse effects of salt stress, namely its osmotic component, on membrane integrity and function. In a comparative proteomic study of salt-treated *A. thaliana* and *T. salsuginea*[22], an augmented abundance of 3-ketoacyl-acyl carrier protein synthase I and phospholipase/carboxyesterase family protein has been found in salt-treated *A. thaliana*. In *T. salsuginea*, an elevated level of a putative long-chain-fatty-acid-CoA ligase involved in fatty acid synthesis and a declined level of a putative glycerophosphodiester phosphodiesterase involved in lipid degradation was found. Increased relative abundance of dihydrolipoamide dehydrogenase and enoyl-ACP reductase (ENR) has been found in salt-treated rice panicles [60]. The changes observed in lipid metabolism in salinity-exposed plants indicate profound changes in cell membrane integrity, composition and function under stress. A short-term decreased relative abundance of monogalactosyl diacylglycerol synthase, an enzyme involved in biosynthesis of galactosylglycerolipids (monogalactosyl diacylglycerol, digalactosyl diacylglycerol), the major components of chloroplast inner envelope and thylakoid system, observed in salt-treated maize chloroplasts [78] and an increased relative abundance of UDP-sulfoquinovose synthase involved in biosynthesis of thylakoid membrane sulfolipids observed in creeping bentgrass [57] indicate profound changes in chloroplast membrane structure and composition in response to salt and osmotic stress.

Changes in lipid-metabolism associated lipid transfer proteins (LTP) and temperature-induced lipocalins have been observed in apoplastic fluid of tobacco cells [53] and tomato radicles [80], respectively, subjected to salinity. Lipid transfer proteins are known to be involved in plant response to pathogens [110] and lipocalins are known to display a protective role against photooxidative damage [111].

### 6.10. Secondary Metabolism

Salinity induces significant alterations also in several metabolic pathways associated with a so-called secondary metabolism. For ion exclusion via transpiration stream, well-developed and lignificated xylem vessels are important. Increased lignification of xylem could be indicated by an elevated level of SAM synthase (SAMS), an important methylation enzyme which plays a crucial role in lignin polymeration [27]. An increased abundance of several proteins (SAMS, xylose isomerase, peroxidases) involved in lignification as a response to salinity has also been observed in salt-treated barley roots [68]. Similarly, an increased relative abundance of caffeic acid 3-*O*-methyltransferase (CCOMT) and caffeoyl-CoA-*O*-methyltransferase (COMT), an enzyme involved in conversion of caffeoyl-CoA to sinapoyl-CoA, an intermediate reaction in the lignification process, has been found in salt-treated foxtail millet [70], barley root [67], tomato root [34] and *Puccinellia tenuiflora* leaves [28]. In contrast, in salt-sensitive soybean, a decreased relative abundance of COMT indicates a reduction in cell wall lignification and a consequent decrease of plant growth [73].

Cyanate represents another compound affected by salinity. Most of cyanate found in plant tissues arises as a byproduct of ethylene biosynthesis. An increased production of cyanate in salt-exposed cells of *Suaeda aegyptiaca* could be expected since an increased relative abundance of cyanase, an enzyme involved in cyanate degradation, has been found out in *S. aegyptiaca* leaves [26]. Interestingly, an increased relative abundance of β-glucosidase dhurrinase, an enzyme involved in hydrolysis of cyanogenic glycosides leading to a release of free hydrogen cyanide, has been found in salt-stressed sorghum [77].

### 6.11. Postranslational Modifications

Differential post-translational modifications (PTMs) of proteins have been found under salt stress with respect to control. Protein reversible phosphorylation plays a crucial role in signalling as well as protein chaperone and enzymatic activities. In salt-treated rice roots, an increased relative abundance of 17 phosphoproteins (e.g., dnaK-type chaperone HSP70, putative GST, small GTP-binding protein OsRac2, mannose-binding RICE lectin) and a decreased relative abundance of 11 phosphoproteins (e.g., putative protein kinase, ATP synthase β chain) has been found [59]. In durum wheat cultivars, differences in phosphorylation pattern of salt-inducible dehydrin DHN5 have been observed [95]. In maize roots, salinity led to phosphorylation of 10 proteins (e.g., fructokinase, UDP-glucosyl transferase BX9, 2-Cys-peroxiredoxin) and dephosphorylation of 6 other e.g., (isocitrate dehydrogenase, CaM, 40S ribosomal proteins; [79]). A salt-induced phosphorylation of several PR10 proteins in peanut has been observed [75]. Due to salinity-associated oxidative stress and stress-associated signalling, oxidative and NO-induced protein PTM can be proposed in salt-treated plants. Tanou *et al.*[83] investigated protein carbonylation (oxidative modification induced by H_2_O_2_) and S-nitrosylation (induced by NO signalling) pattern in salt-treated citrus leaves with or without H_2_O_2_ or sodium nitroprusside (a NO releasing chemical) pretreatment and suggested an important role of protein carbonylation and S-nitrosylation patterns in salinity response of citrus. In their study, a strong increase in the level of carbonylated (ADH, chaperonin 60 subunit α, glycolytic enzymes, HSP70, RubisCO LSU, subunits of chloroplast and mitochondrial ATP synthase F1, mitochondrial processing peptidase) and nitrosylated proteins (actin, annexin, ENO, GAPDH, GST, HSPs, RubisCO activase, RubisCO LSU, PGK, TPI, SOD, peroxiredoxin, tubulin, several eIF and eEF) has been found under salt stress. The results denote that salinity does not only affect protein relative abundance, but also their PTM and thus their function and catalytic activity.

The observed differences at proteome level regarding not only changes in protein relative abundance, but also changes in PTM, protein interactions and protein activity significantly determine differences between salt-sensitive and salt-tolerant plants observed at metabolite and physiological (functional) levels.

## 7. Conclusions and Future Perspectives

Salinity profoundly affects various aspects of plant cell structure and metabolism. Salinity with its osmotic and ionic aspects induces cellular adjustment associated with a profound reorganization of cell structure and metabolism. Proteins play a major role in salt stress acclimation and plant cellular adjustment since proteins are involved in a wide array of cellular processes associated with salt acclimation including signalling, regulation of gene expression and protein metabolism, energy metabolism, redox metabolism, osmolyte metabolism, defence-related proteins, mechanical stress-related proteins, phytohormone, lipid and secondary metabolism. Comparative proteomic analysis has enabled the researchers to identify differentially abundant proteins in genetically related plant materials revealing differential stress tolerance [23,112]. Besides changes in protein relative abundance, changes in protein posttranslational modification (PTM) pattern as well as protein activity have been observed under salinity.

The differences between the sensitive (glycophytes) and the tolerant (halophytes) plants at protein level represent only a piece of a complex adaptation to salinity found in salt-tolerant plants. It is becoming evident that the differences in salinity response at protein level are conferred by differences at genome and transcriptome level including a higher gene copy number and an altered structure of promoter sequences in salt-tolerant plants with respect to salt-sensitive ones. The differences at genome level are reflected at transcript and protein levels resulting in differential transcript and protein expression levels between susceptible and tolerant plants. Correspondingly to differences at genome level (changes in gene copy number and promoter structure and organization), comparative transcriptomic and proteomic studies have revealed that differences at transcript and protein levels regarding several stress-induced genes between salt-tolerant and salt-sensitive plants can be detected even under control (non-stressed) conditions [19,21] similarly to situation reported in other stresses (e.g., COR/LEA proteins under cold; [113]). The changes at protein level reveal also a crucial impact on plant cell structure and metabolism. Regarding the functional aspects of the changes in proteome composition, not only alterations in protein relative abundance, but also changes in protein posttranslational modification (PTMs) and protein-protein interactions deserve to be studied. Taken together, the results from comparative proteomic studies represent an important step to understanding plant salt response processes as a whole. However, the results should be validated and combined with results from genomic, transcriptomic, metabolomic, functional and physiological studies to unravel the complexity of plant salinity responses.

## Supplementary Information



## Figures and Tables

**Table 1:** Examples of adaptations of tolerant halophytic plants to salinity. Ref.: references.

Level of study	Salinity adaptation	Ref.
Genomic	Gene duplication (increased gene copy number) and promoter adaptation of several salinity-responsive genes (transcription factors: Myb24, ATPase AVP1, ion transporters: SOS1, NHX; ABC)	[18]
Transcriptomic	Enhanced constitutive expression of several salinity-responsive transcripts (SOS1, SOD, P5CS, GS, INPS, cytochrome P450, heat shock protein ) Hsc70-3, antifugal protein PDF1.2)	[19–21]
Proteomic	Enhanced abundance of several stress- and defence-related proteins (LEA, redox, PR), ion transporters, protective proteins involved in activation of photosynthesis (D2 protein) and protein biosynthesis, activation of biosynthesis of protective compounds (lignin)	[22–27]
Metabolomic	Alterations in carbohydrate metabolism—activation of catabolism (glycolysis, Krebs cycle, starch degradation), enhanced biosynthesis of organic osmolytes, phenolic compounds, lignin)	[20,22, 28,29]

	**Osmotic effect of salinity**	
Functional (physiological) level	- osmotic adjustment (accumulation of low-molecular organic osmolytes and proteins—LEA proteins)	[21,30–33]
	- adjustment to mechanical stress (increased cell wall lignification, accumulation and oligomerization of several coat proteins in plasmamembrane of *Dunaliella salina*)	[27,28, 32,34]
	**Ion-related effects of salinity**	
	- salt ion exclusion (increased abundance and activity of plasma membrane ion transporters (SOS1), increased lignification of xylem vessels (long-distance transport of excluded salt ions via transpiration stream— *Salicornia europaea*)	[27]
	- salt ion intracellular compartmentation (salt import into vacuoles— an enhanced abundance of tonoplast ion transporters NHX, support of ion transport - H^+^-ATPase and FBP aldolase activity)	[24,35]

**Table 2:** A list of major protein functional groups revealing differential protein abundance under salinity in glycophytes and halophytes. References to the papers providing cited data are given in brackets.

Protein functional group	Glycophytes		Halophytes	
	Increased protein	Decreased protein	Increased protein	Decreased protein
Signalling	Annexin [22], calmodulin, OsRPK1 [91], calreticulin [51,84] MAPK [56,76], large GTP binding protein; β subunit of heterotrimeric G protein, OsRac2 [22,23,51,52,59], 14-3-3 [23,56,79,89]	Ras GTPase, 14-3-3 [52]	Annexin [22,27], β subunit of heterotrimeric G protein [23], phototropin [89], alpha-SNAP [28]	CBL-interacting protein kinase [27], 14-3-3 [28]
Gene expression regulation, cell growth and division	NAC-α, HB1B, OSAP1 RNA helicase [30,62,65,80]	NAC-α, polyA-binding protein [51]	CRT/DREB [16], Cell division protein fts homolog [89]	SKP1-like protein [89], MorR homolog [90], TF APFI [27]
Protein biosynthesis and degradation	eIF, eEF, eIF5A3, ribosomal proteins L12, L31, S29 [22], Proteasome 20S [60], 26S [70], GS [62]	eIF-4E2, eEF [52], L10, L12, S3a, S12 [84], L29, S5 [22], GS [81]	eIF3A, S7, S24, S15a [22] Alpha type 6, Beta type 1, GS [22,27]	20S, Proteasome subunit alpha type 2, 4, 6 [28]
Protein folding	DnaK chaperone, HSP70, small HSP [80], RubisCO binding protein subunit β (CPN60-β) [30,65,66]	HSP90 [57]	Small HSP [88], HSP70 [89], CPN60-α [28], HSP60 [27]	HSP90 [28]
Photosynthesis	23 kDa (PsbP), ferredoxin-NADPH reductase [78], OEE2 [65,66], RubisCO activase [61,65,92], TPI, GAPDH, Glucose-6-P dehydrogenase [56,58,73]	LHC, PC, OEE1, OEE2, RubisCO LSU and SSU, RubisCO activase [22,56,71,73,81], carbonic anhydrase; GAPDH, SBP, PGK, PRK, TK [61,71,92]	LHC, OEE2, RubisCO LSU and SSU, RubisCO activase [16,89], D2 protein PSII [26], CP24, CP47, PSI subunit IV [16], carbonic anhydrase; SBP, PGK [29,87]	LHC, OEE1, RubisCO LSU and SSU, RubisCO activase [28], PsbO [90], SBP [26,86]
Respiratory pathway and sucrose matabolism	FBP aldolase, GAPDH, TPI, ENO [61], Succinyl-CoA ligase β subunit, MDH (NAD), cytochrome c oxidase subunit 6b-1 [22], ATP synthase CF1β,δ,ɛ ; ADK, NDPK1 [22,63,74,78], ADH [73]	ATP synthase CF1α,β-3, mtATP β-3 [77]	FBP aldolase [35], ATP synthase CF1β,ɛ, ADK, NDPK1 [86], Fructokinase-like protein [89], GAPDH, MDH [27]	ATP synthase CF1α,β [89], MDH [27], sucrose synthase [28]
Redox metabolism	GST, APX, Cu/Zn-SOD, Mn-SOD, PRX, Trx *h*, protein disulfide isomerase (practically all papers)	APX, MDAR [51]	GST, APX, Cu/Zn–SOD, 2-Cys PRX, MDAR (practically all papers)	APX chain A [86], MDAR [27], GST-I, Thioredoxin [28]
Metal binding proteins	Ferritin, IDI2, IDS2, IDS3 [68], magnesium chelatase [66], metallothionein [70]		Ferritin, Voltage-dependent anion channel protein [27], transferrin-like [31], FutA1, FutA2 [90]	Ferritin [27]
Defence-related proteins	PR5, PR10 [22,25,67,72,74,75,82,84,92], β-1,3- glucanase, cyanogenic β-glucosidase dhurrinase [77], (1–3)-b-Glucanase GV [68], Jacalin lectin, mannose-binding RICE lectin [59], lectin-like [56,76], Germin-like [51,65], SAMS [68]	Lectin-like [72], 23 kDa jasmonate-induced protein, F23N19.10 stressinducible protein [68], SAMS [73], TSI-1 [34]	NBS-LRR disease resistance [27], SAMS [27,28], Lipocalin-like protein [89]	Disease resistance protein [27]
Transport	PM H^+^- ATPase, V-ATPase subunit β (VHA-B), PPase, NHX [22–24,51,57,82], ABC [23]	V-ATPase subunit β (VHA-B) [34]	HKT, V-ATPase subunit β (VHA-B), PPase, NHX [22,23], ABC [86,90]	V-ATPase subunit β [28], Putative porin [90]
Cytoskeleton-related	Profilin [65], β-tubulin [22,51]	Tubulin, actin [83]	Profilin [26]	
Structural proteins	Myosin VIII, remorin [91], OSR40c1, β-d-glucan exohydrolase, GRP [57,60,82]	XET [84]	PM coat proteins [32], cellulose synthase [16]	
Lipid metabolism	3-ketoacyl-acyl carrier protein synthase I, phospholipase/carboxyesterase family protein, dihydrolipoamide dehydrogenase, ENR [60], UDP-sulfoquinovose synthase [57]	Monogalactosyl diacylglycerol synthase [78]	Long-chain-fatty-acid-CoA ligase [22]	Glycerophosphodiesterase [22]
Phytohormone metabolism	JA biosynthesis (AOC, LOX) [22,51]		ABA biosynthesis (NCED) [19], GA biosynthesis (DWARF3) [23,64]	
Lignin biosynthesis	COMT, CCOMT [34,67,70]	COMT, CCOMT [73]	COMT, CCOMT [27,28]	

## Abbreviations

2D-DIGE: two-dimensional difference gel electrophoresis
ABA: abscisic acid
ACP: acyl carrier protein
ADH: alcohol dehydrogenase
ADK: adenylate kinase
ALDH: betaine aldehyde dehydrogenase
AOC: allene oxide cyclase
AOX: alternative oxidase
APX: ascorbate peroxidase
CAB: chlorophyll *a*/*b* binding protein
CaM: calmodulin
CAT: catalase
Chl: chlorophyll
CK: cytokinin
CCOMT: caffeic acid 3-*O*-methyltransferase
CMO: choline monooxygenase
COMT: caffeoyl-*O*-methyltransferase
DHAR: dehydroascorbate reductase
DHN: dehydrin
DNPH: dinitrophenylhydrazine
eEF: eukaryotic elongation translation factor
eIF: eukaryotic initiation translation factor
ENO: enolase
ENR: enoyl-ACP reductase
FBP: fructose bisphosphate
FFZE: free flow zonal electrophoresis
GA: gibberellin
GAPDH: glyceraldehyde-3-phosphate dehydrogenase
GB: glycine betaine
GRP: glycine-rich protein
GS: glutamine synthetase
GST: glutathione-*S*-transferase
HK: histidine kinase
HKT: high-affinity potassium transporter
HSP: heat shock protein
IDI: iron deficiency-induced
IDS: iron deficiency-specific
INPS: l-*myo*-inositol-1-phosphate synthase
JA: jasmonic acid
LEA: Late embryogenesis abundant
LHC: light-harvesting complex
LOX: lipoxygenase
LRR: leucine-rich repeat
LTP: lipid transfer protein
MAPK: mitogen-activated protein kinase
MDAR: monodehydroascorbate reductase
MDH: malate dehydrogenase
MRL: mannose-binding RICE lectin
NCED: 9-cis-epoxy-dioxygenase
NDPK: nucleotide diphosphate kinase
NHX: Na^+^/H^+^ exchanger
OEC: oxygen evolving complex
OEE: oxygen evolving enhancer
P5CS: pyrroline-5-carboxylate synthase
PA: phosphatidic acid
PC: plastocyanin
PDH: proline dehydrogenase
PEPCase: phosphoenolpyruvate carboxylase
PGK: phosphoribulokinase
PH: phytohormone
PK: protein kinase
PLC: phospholipase C
PLD: phospholipase D
PPase: pyrophosphatase
PPP: pentose phosphate pathway
PR: pathogenesis-related
PRK: phosphoribulokinase
PRX: peroxiredoxin
PS: photosystem
PS RC: photosystem reaction center
PTM: posttranslational modification
ROS: reactive oxygen species
RubisCO: ribuloso-1,5-bisphosphate carboxylase/oxygenase
RubisCO LSU: RubisCO large subunit
RubisCO SSU: RubisCO small subunit
SA: salicylic acid
SAM: *S*-adenosylmethionine
SAMS: *S*-adenosylmethionine synthetase
SBP: sedoheptulose-1,7-bisphosphate
SOD: superoxide dismutase
SOS: salt overly sensitive
SUS: sucrose synthase
TCA: tricarboxylic acid cycle
TPI: triose phosphate isomerase
TPR: tetratricopeptide repeat
Trx: thioredoxin
V-ATPase: vacuolar ATPase
VDAC: voltage dependent anion channel
XET: xyloglucan endotransglycosylase
