# Supplementary material for: Protein Contribution to Plant Salinity Response and Tolerance Acquisition
Source: Int J Mol Sci. 2013 Mar 26;14(4):6757–89. doi: 10.3390/ijms14046757 (PMC3645664; doi:10.3390/ijms14046757)
Supplement: Supplementary file 1 [file ijms-14-06757-s001.doc]

**Supplementary Information**

**Table S1.** A list of proteomic studies focused on plant salinity response in glycophytes (**A**) and halophytes (**B**). Plant material, salinity treatments and methods used for protein detection and identification, the number of differentially abundant proteins found, the major results gained by the study (identified proteins and their possible functions in stress) and the corresponding reference are given. DP- differentially abundant proteins, IdP: identified proteins, Ref,: reference.

| **Organism** | **NaCl treatment/Method** | **DP (IdP)** | **Major results (identified proteins and their possible functions)** | **Ref.** |
| --- | --- | --- | --- | --- |
| **A. Glycophyte** | | | | |
| *Arabidopsis thaliana* Col-0 cell suspension culture | 200 mM for 6 h, 4 h recovery/2D-DIGE,  MALDI-TOF | 266 (75) | Up: detoxyfing enzymes (SOD, peroxiredoxin, GST; glycolytic enzymes; Down: protein biosynthesis | [39] |
| Col-0 root | 150 mM (6, 48 h)/2DE  LC-MS/MS | 215 (85) | Up: ROS scavenging enzymes; Down: energy metabolism  (glycolysis, mitochondrial respiration, pentose phosphate pathay) | [38] |
| Rice (*Oryza sativa*) Cvs Nipponbare, IR36,  Pokkali: root, leaf sheath | 50, 100, 150 mM (6, 24,  48 h)/2DE Edman sequencing | 8 (5) | Up: OEE2, FBP aldolase, SOD | [45] |
| Cv Nipponbare seedlings:  3rd leaf | 130 mM (4 d)/2DE  nanoESI-LC-MS/MS | 33 | Up: RubisCO, RubisCO activase, FBP aldolase Down: some isoforms of RubisCO, FBP aldolase, TK | [48] |
| Cv Nipponbare: seedling root | 150 mM (24, 48, 72 h)/2DE MALDI-TOF | 54 (10) | Up: UDP-glucose pyrophosphorylase, cytochrome *c* oxidase subunit 6b-1, GS, putative actin-binding protein, NAC-1α | [49] |
| IR651: panicle | 50 mM (7 d) followed by  75 mM (5 d)/2De  MALDI-TOF/TOF | 29 | Up: GSH-DHAR, thioredoxin h, ENR, profilin, STI-1, GRP | [47] |
| Cv Nipponbare: root phosphoproteome | 150 mM (10, 24 h)/2DE MALDI-TOF/TOF;  Pro-Q Diamond | 28 (17↑11↓) | Up: GST, ribosomal protein S29, dnaK-type molecular chaperone HSP70, MRL Down: GAPDH, ATP synthase β chain | [46] |
| Barley (*Hordeum vulgare*) OUK305 (tolerant), OUI743 (sensitive): root | 200 mM (5 d)/2DE  LC-MS/MS | 6 | Up: APX, DHAR, GST; PR10; COMT | [56] |

**Table S1.** *Cont.*

| **Organism** | **NaCl treatment/Method** | **DP (IdP)** | **Major results (identified proteins and their possible functions)** | **Ref.** |
| --- | --- | --- | --- | --- |
| Cvs Morex (tolerant) and Steptoe (sensitive): root | 100 and 150 mM  (13 d)/2DE MALDI-TOF  or nanoLC-ESI-Q-TOF MS/MS | 39 (26; 5↑16↓) | Up: IDS2, IDS3, IDI2 – phytosiderophore biosynthesis (Fe uptake); SAMS, peroxidase, APX, (1–3)-b-Glucanase GV Down: 23 kDa jasmonate-induced protein, F23N19.10  stress-inducible protein, Fructokinase 2 | [57] |
| Oregon Wolfe Barley mapping population—selected DH lines—grain | 1.5, 2, 2.5% (20 d)/2DE MALDI-TOF/TOF | 11 (3↑3↓) | Upregulation of 6-phosphogluconate dehydrogenase and glucose/ribitol dehydrogenase in tolerant lines versus sensitive ones Decreased abundance of HSP70, Putative elongation factor 1b, Translationally controlled tumour protein homolog in tolerant lines | [58] |
| Cvs Afzal (tolerant) and L-527 (sensitive) – leaf | 300 mM (24 h)/2DE  MALDI-TOF/TOF | 117 (22) | Up (both): PRK, SBP; FBP aldolase; magnesium chelatase;  2-Cys PRX Down (sensitive): OEE2, PC | [55] |
| Cvs Afzal (tolerant) and L-527 (sensitive) – leaf | 300 mM (21 d)/2DE  MALDI-TOF/TOF | 44 (43↑1↓) | Up (both): RubisCO activase, OEE2, ribosomal protein S1, NAC, profilin, GLP, NAC; sensitive line 527 –Trx, DHAR; tolerant – PAO (polyamine oxidase) | [54] |
| Common wheat (*Triticum aestivum*) Cvs Wyalkatchem (tolerant), Janz (sensitive) – shoot mitochondrial fraction | 200 mM (4d)/2D-DIGE  LC-MS/MS | 192 (68) | Up: Mn-SOD; VDAC; AOX; NDPK (a higher increase in tolerant cultivar than in sensitive one) | [51] |
| Durum wheat (*Triticum turgidum* ssp*. durum*) Cv Ofanto - leaf | 100 mM (2 d)/2DE  MALDI-TOF | 38 (28↑10↓) | Up: RubisCO activase, RubisCO binding protein; glycolytic enzymes (GAPDH, TPI), SAMS; APX, Cu-Zn SOD; carbonic anhydrase; LEA/RAB Down: OEE1, RubisCO SSU, Calvin cycle enzymes (PRK, PGK, FBP aldolase), ATP synthase CF1 α, β-glucosidase | [50] |
| Sorghum (*Sorghum bicolor*) Csv-17 - leaf | 200 mM (96 h)/2DE  MALDI-TOF/TOF | 18↑3↓ | Up: kinases (lectin-like protein kinase, Ser/Thr kinase),  β-1,3-glucanase, ROS scavenging enzymes (GST, peroxidase) | [66] |

**Table S1.** *Cont.*

| **Organism** | **NaCl treatment/Method** | **DP (IdP)** | **Major results (identified proteins and their possible functions)** | **Ref.** |
| --- | --- | --- | --- | --- |
| MN1618 - leaf | 100 mM (14 d)/2DE  MALDI-TOF/TOF | 118 (55) | Up: RubisCO large subunit, eEF1-δ; cyanogenic β-glucosidase dhurrinase Down: OEE1, Calvin cycle enzymes (PRK, FBP aldolase, Ru5P isomerase), ATP synthase CF1α | [67] |
| Maize (*Zea mays*) Hybrid SR12 – chloroplast fraction | 25 mM (1, 2 and 4 h)/2DE MALDI-TOF | 20 | Up: PSII associated protein PsbP, FtsH-like; ferredoxin NADPH oxidoreductase, protoporphyrinogen IX oxidase; ATP synthase CF1δ Down: ATP synthase CF1α,ε | [68] |
| Hybrid SR12 – root phosphoproteome | 25 mM (1 h)/2DE  MALDI-TOF/TOF; PhosTag | 10↑6↓ | 10 proteins salinity-phosphorylated: fructokinase, UDP-glucosyl transferase BX9, 2-Cys-peroxiredoxin 6 proteins salinity-dephosphorylated: isocitrate dehydrogenase, CaM, maturase, 40 S ribosomal protein | [69] |
| Foxtail millet (*Setaria italica*) Cv. Prasad - seedling | 100, 150, 200 mM (7 d)/2DE MALDI-TOF/TOF | 29 | Up: photosynthesis-related (PSI subunit IV), ATP synthase F1 β subunit; GS; matallothionein; lignin biosynthesis (CCOMT) | [59] |
| Creeping bentgrass (*Agrostis stolonifera*) – Cv. Penn-A4 (tolerant), Penncross (sensitive) – root, leaf | 2 dS (2 d) + 4 dS (2 d) + 6 dS (2 d) + 8 dS (2 d) +10 dS  (28 d)/2D-DIGE  MALDI-TOF/TOF | 148 (106) leaf 40 (24) root | Up (tolerant): NDPK, V-ATPase (root); CAT, GST,  UDP-sulfoquinovose synthase, β-glucan exohydrolase (leaf);  Down: HSP90, HSP81-1, pentameric polyubiquitin (leaf and root) | [44] |
| Canola (*Brassica napus*) – Cv. Hyola 308 (tolerant), Sarigol (sensitive) – leaf | 175, 350 mM (21 d)/2DE MALDI-TOF/TOF | 44 in Hyola 31 in Sarigol | Up in both: Cu/Zn-SOD, 2-peroxiredoxin Up in tolerant (Hyola 308) only: RubisCO SSU, RubisCO activase; eIF5A | [60] |
| Sugar beet (*Beta vulgaris*) Cv Evita – root, shoot plasma membrane (PM) fraction | 125 mM (7 d)/Q-TOF MS | (4↑2↓) shoot 3↑ root | High constitutive level of PM ion transporters (VDAC, H^+^-ATPase) Down: ferredoxin-NADP reductase, aminomethyltransferase | [61] |
| Peanut (*Arachis hypogaea*) Cv. JL24 – callus -phosphoproteome | 50, 200 mM /2DE  ESI-Q-TOF MS/MS;  Pro-Q Diamond | 24 | Upregulation and phosphorylation of several PR10 proteins | [65] |

**Table S1.** *Cont.*

| **Organism** | **NaCl treatment/Method** | **DP (IdP)** | **Major results (identified proteins and their possible functions)** | **Ref.** |
| --- | --- | --- | --- | --- |
| Pea (*Pisum sativum*) – Cv. Cutlass – root | 75, 150 mM (7 d)/ 2DE  ESI-Q-TOF MS/MS | 35 | Up: SOD, NDPK, PR10 | [64] |
| Grasspea (*Lathyrus sativus*) var. LP-24 - leaf | 500 mM (12, 24, 36 h)/ 2DE | 48 | Up: ADH, FBP aldolase; SOD, GPX, thioredoxin *m*,  2 Cys-peroxiredoxin; MAPK, NCED, 14-3-3 Down: RubisCO LSU, OEE3 (enhanced degradation) | [43] |
| Soybean (*Glycin max*) Cv. Enrei – root, hypocotyl | 100 mM (3 d)/ 2DE  ESI-Q/TOF-MS/MS | 20 (4↑3↓) | Up: LEA, β-conglycinin, elicitor peptide three precursor,  basic/helix-loop-helix protein Down: protease inhibitor, lectin, stem 31-kDa glycoprotein precursor | [62] |
| Cv. Enrei – leaf, hypocotyl, root | 40 mM (7 d)/2DE  MALDI-TOF | 19-leaf, 22-hypocotyl, 14-root | Up: ADH (anaerobic metabolism); kinesin, 20 kDa chaperonin  (co-chaperone with cpn60) Down: RubisCO activase, 50S ribosomal subunit protein,  caffeoyl-CoA-O-methyltransferase (lignin biosynthesis) | [63] |
| Potato (*Solanum tuberosum*) Cvs Concord (sensitive), Kennebec (tolerant) – shoot | 90 mM (28 d)/2DE Edman sequencing | (16↑22↓) | Up: Ca transport and signalling (annexin, calreticulin), stress response (stromal HSP, TSI-1, osmotin-like) Down: photosynthesis (OEE1, RubisCO SSU), protein biosynthesis (50S ribosomal protein, GS) | [72] |
| Tomato (*Lycopersicon esculentum*) Cvs F144 (sensitive), Patio (tolerant) – hypocotyl, radicle | 120 mM (7 d)/2DE ESI-LC-MS/MS | 23 | Up: NACα, small HSP (HSP17.6, HSP17.8, HSP20.1), SOD, cAPX, ferritin, temperature-induced lipocalin Down: RubisCO, ATP synthase (mitochondrial), | [70] |
| Cvs Levovil (tolerant), Roma, Supermarmade (intermediate), Cervil (sensitive) - root | 100 mM (14 d)/2DE MS/MS | 48 genotype and salinity differences | Up: HSP90, APX, GS (tolerant); cell-wall biosynthesis enzymes (caffeoyl-CoA-O-methyltransferase 6, glucose:protein transglucosylase like SIUPTG1) Down: V-ATPase subunit B; TSI-1 | [71] |
| Cucumber (*Cucumis sativus*) Cv Jinchun No 2 – seedling root | 50 mM (7 d)/2DE MALDI-TOF/TOF,  LC-ESI-MS/MS; RT-PCR (transcript) | 34 (29 - 21↑8↓) | Up: V-ATPase subunit A, Cu/Zn-SOD, peroxiredoxin, HSP70, profilin, GRP Down: mtATP synthase, CAT-3, APX | [73] |

**Table S1.** *Cont.*

| **Organism** | **NaCl treatment/Method** | **DP (IdP)** | **Major results (identified proteins and their possible functions)** | **Ref.** |
| --- | --- | --- | --- | --- |
| Tobacco (*Nicotiana tabacum*) Cv Petit Havana – leaf apoplast fluid | 100 mM (20 d)/2DE  LC-MS/MS | 20 | Up: peroxidase, LTP, germin-like, chitinase PR-P, α-galactosidase | [40] |
| Cv Wisconsin - leaf | 150 mM (2 d)/2DE Edman sequencing | 18 | Up: RubisCO LSU, RubisCO SSU, OEE2 Down: HSP70 | [41] |
| Citrus (*Citrus aurantium*) – leaf; Protein carbonylation and  S-nitrosylation | 150 mM (16 d)/2DE  nano-LC-Q-TOF Carbonylation: DNPH treatment and anti-DNP antibody (W-blot) S-nitrosylation: biotin-switch method | 40  49 | Carbonylation: RubisCO LSU, subunits of chloroplast and mitochondrial ATP synthase F1, glycolytic enzymes, ADH, HSP70, chaperonin 60 subunit α, mitochondrial processing peptidase S-nitrosylation: RubisCO LSU, RubisCO activase, GAPDH, ENO, PGK, TPI, GST, SOD, peroxiredoxin, glutaredoxin, tubulin, actin, annexin, HSPs, several eIF and eEF | [74] |
| Grapevine (*Vitis vinifera*) Cvs Chardonnay (tolerant), Cabernet Sauvignon (sensitive) - shoot | (10 mM to 250 mM – 16 d)/2DE MALDI-TOF/TOF | 202 | Chardonnay: upregulation of ribosomal protein L39 Downregulation of XET Cabernet Sauvignon: upregulation of mtPRX, bHLH, PR10 | [76] |
| Cv Razegui – leaf, stem, root | 100 mM (15 d)/2DE Edman sequencing | 48 (32↑9↓;  7 new) | Up: PR10 | [75] |
| Poplar (*Populus cathayana*) male and female plant leaves | 75, 150 mM (28 d)/2DE  ESI-Q-TOF MS/MS |  | Male: higher abundance of RubisCO activase, RubisCO LSU binding protein subunit β, OEE2, APX, GST class phi than in female | [42] |
| **B. Halophyte** | | |  |  |
| *Aeluropus lagopoides* leaf | 150, 450, 600, 750 mM (10 d)/ 2DE nanoLC-MS/MS | 83 (60↑53↓) | 24 spots contained more than one protein! Up: C4 photosynthesis (PEPCase; RubisCO activase), energy production (ATP synthase α), amino acid biosynthesis, transport (RanGTP), defense (HSP70, GST, 2-cys PRX) Down: RubisCO LSU, Calvin cycle enzymes (FBP aldolase, TK) | [95] |
| *Aster tripolium* - leaf | 450 mM, elevated CO_2_ (520 ppm)/2DE MALDI-TOF | (5↑) | Up (salinity plus elevated CO_2_): LHC chlorophyll *a*/*b* binding protein, V- ATPase subunit β; HSP20, GST, SOD | [81] |

**Table S1.** *Cont.*

| **Organism** | **NaCl treatment/Method** | **DP (IdP)** | **Major results (identified proteins and their possible functions)** | **Ref.** |
| --- | --- | --- | --- | --- |
| *Bruguiera gymnorhiza* root, leaf | 500 mM (1,3,6,12, 24 h; 3, 6, 12 d)/2DE LC-MS/MS | 6 (2↑) | Up: FBP aldolase, osmotin-like protein | [83] |
| *Mesembryanthemum crystallinum* – leaf – microsomal fraction | 200 mM (7 d)/2D-DIGE free flow zonal electrophoresis (FFZE) ESI LTQ-Orbitrap MS | 8 (6↑) | Up: V-ATPase subunit β (VHA-B), glycolytic enzymes FBP aldolase, ENO; interaction between FBP aldolase and VHA-B proven to stimulate V-ATPase activity | [80] |
| *Puccinellia tenuiflora* - leaf | 50, 150 mM (7 d)/2DE  ESI-Q-TOF | 188 (28↑79↓) | Up: photorespiration, AAA ATPase, PPP and TCA cycle enzymes; aminopeptidase N, oligopeptidase A; CCOMT, tocopherol cyclase  Down: LHC, RubisCO LSU and SSU, carbonic anhydrase, Calvin cycle enzymes (PRK), cytosolic HSP90, protein disulfide isomerase precursor | [82] |
| *Salicornia europaea* shoot | 200 mM shock (12, 24, 72 h), 200, 600, 800 mM adaptation (21 d)/2DE MALDI-TOF/TOF | 196 (111; shock-78↑23↓; 85↑23↓) | Up: photosynthesis (RubisCO activase, OEE, RubisCO LSU), energy production (ATP synthase), osmolyte biosynthesis (CMO, SAMS), ion transport (V-ATPase, VDAP), cytoskeleton (profilin), lignification (SAMS, xylose isomerase), GST 6, SOD, MDAR, PRP 10a, DNA topoisomerase II, annexin Down: MDH, SAMS2, MDAR, RubisCO LSU, Ferritin, Transcription factor APFI | [84] |
| *Suaeda aegyptiaca* leaf | 150, 300, 450, 600 mM  (30 d)/2DE LC-MS/MS | 102 (25↑2↓) | Up: photosynthesis (D2 protein PSII), glycine betaine synthesis (CMO, SAMS), cytoskeleton (profilin), cyanose, DHAR, SOD, glutathion peroxidase Down: SBP, RubisCO SSU | [78] |
| *Suaeda salsa* leaf | 100, 200 mM (21 d)/2DE MALDI-TOF; combination with heat-shock | 147 (57; 13↑17↓) | Up: photosynthesis (RubisCO activase, LHCII chlorophyll *a*/*b* binding protein), energy production and conservation (ATP synthase, ADK, NDPK), transport (V-ATPase, ABC1), CMO Down: RubisCO LSU, GAPDH, serine hydroxymethyltransferase, APX chain A, SBP | [79] |

**Table S1.** *Cont.*

| **Organism** | **NaCl treatment/Method** | **DP (IdP)** | **Major results (identified proteins and their possible functions)** | **Ref.** |
| --- | --- | --- | --- | --- |
| *Dunaliella salina* Plasma membrane, chloroplast- and cytosol-soluble fraction | 0.5, 3 M (several weeks)/2DE LC-MS/MS | 76 (45↑) | Enhancement of photosynthesis, Calvin cycle, PPP, photorespiration, ATP production; up: eIF3, tubulin | [86] |
| Plasma membrane | 0.5, 3 M (several weeks)/ 2D-BN/SDS LC-MS/MS | 35 (55; 20↑2↓) | 20 spots contained more than one protein! Up: mechanical protection (surface coat lipoproteins), ion transport (H^+^-ATPase), chaperone (HSP70, HSP90), antioxidants (Fe-SOD, GST) Down: Lysophospholipase A | [87] |
| *Synechocystis* PCC6803 Plasma membrane | 684 mM (6, 8 d)/2DE MALDI-TOF | 25 (106; 20↑5↓) | Up: transport (ABC transporter), iron-binding (FutA1, FutA2) Down: MorR homolog, PSII subunit PsbO, putative porin | [88] |
| *Physcomitrella patens* - gametophyte | 250,300, and 350 mM (72 h)/ 2DE, LC-MS/MS | 65 (49↑16↓) | Up: photosynthetic proteins (RubisCO LSU, RubisCO SSU, LHC chl *a*/*b* binding protein); HSP70; Fructokinase-like protein, ROS scavenging enzymes (2-Cys peroxiredoxin, LOX, cytochrome P450 monooxygenase); phototropin, 14-3-3 Down: ATP synthase, SKP1-like protein | [85] |

**Table S2.** A list of comparative transcriptomic and proteomic studies carried out on related plant species with contrasting level of salinity tolerance (a glycophyte and a halophyte), the treatments and methods used and the major differences found at transcript or protein level in response to salinity. Ref.: reference.

| **NaCl treatment/Method** | **Major results—transcripts or proteins revealing a  differential response to salinity between a glycophyte and a halophyte** | **Ref.** |
| --- | --- | --- |
| *Arabidopsis thaliana* Col-0 *vs*. *Thellungiella salsuginea* Shandong | |  |
| 250 mM (2 h)/full-length Arabidopsis cDNA microarray (ca 7000 genes) | Higher constitutive expression of stress-responsive genes in *Thellungiella* (*SOS1*, *Fe-SOD*, *P5CS*, *PDF1.2*, P-protein, *AtNCED*, β-glucosidase) | [21] |
| 150 mM (*A. thaliana*), 250 mM (*T. salsuginea*); (3, 24 h)/25,000 cDNA element Arabidopsis microarray, qPCR | Higher constitutive expression of genes involved in ABA synthesis and ABA responsiveness, LTP, HSP, GDSL lipases, cell-wall synthesis genes in *Thellungiella* | [34] |
| 100, 250, 500, 750 mM/qPCR | Higher constitutive expression of *SOS1* and lower constitutive expression of *PDH* in *Thellungiella* | [35] |
| 50, 150 mM (5 d) - leaves/2DE MALDI-TOF/TOF; iTRAQ LC-MS/MS | *Arabidopsis*: 88 differentially abundant proteins, 79 identified; upregulation of respiratory enzymes; JA metabolism (AOC2, LOX2), ion transport (V-ATPase), ROS and defense (GST, PR5); downregulation of RubisCO activase, ribosomal proteins (S5, L29) *Thellungiella*: 37 differentially abundant proteins, 32 identified; upregulation of RubisCO activase; ROS (APX), ribosomal proteins (S7, S15A, S24); Upregulation of salt-responsive transcripts HSC70-3, P5CS, FtsH protease, eIF3A | [36] |
| Common wheat (*Triticum aestivum*) cv. Jinan 177 *vs*. *Triticum aestivum/Thinopyrum ponticum* introgression hybrid Shanrong 3 | | |
| 200 mM (24 h) - root/2DE MALDI-TOF/TOF; Affymetrix wheat GeneChip | *T. aestivum* Jinan 177: 73 differentially abundant proteins; induction of redox (putative reductase), transport (ABC, EXO70) *T.aestivum/T. ponticum* Shanrong 3: 66 differentially abundant proteins; induction of energy metabolism (mtATP synthase), ion transport (V-ATPase subunit E), gibberellin biosynthesis (DWARF3) Both: induction of small G proteins, 14-3-3 proteins | [53] |

**Table S2.** *Cont.*

| **NaCl treatment/Method** | **Major results—transcripts or proteins revealing a  differential response to salinity between a glycophyte and a halophyte** | **Ref.** |
| --- | --- | --- |
| 200 mM (24 h) – root, leaf/2DE MALDI-TOF/TOF | *T. aestivum* Jinan 177: 93 differentially abundant proteins in roots and 65 in leaves; enhanced ethylene receptor (ETR1), enhanced degradation of RubisCO subunits than in Shanrong 3 *T.aestivum/T. ponticum* Shanrong 3: higher levels of several ROS scavenging enzymes (GST F4, POD, SOD), constitutively higher abundance of V-ATPases (V-ATPase subunit E), enhanced accumulation of CP24 (PSII) protein than in Jinan 177 | [52] |
| Rice (*Oryza sativa*) Cvs Pokkali (tolerant), IR64 (sensitive) *vs*. Wild rice (*Porteresia coarctata*) | |  |
| 200, 400 mM (72 h)/ 2DE  MALDI-TOF – 20 proteins in leaves | *P. coarctata*: higher levels of photosynthesis-related proteins (33 kDa Mn-stabilizing OEC, CP47, RubisCO, RubisCO activase); HSP70; cellulose synthase; *myo*-inositol-1-phosphate synthase (INPS), energy-saving enzymes sucrose synthase (SUS) than in *O. sativa* | [16] |

© 2013 by the authors; licensee MDPI, Basel, Switzerland. This article is an open access article distributed under the terms and conditions of the Creative Commons Attribution license (http://creativecommons.org/licenses/by/3.0/).
